# Dietary Phosphorus Reduced Hepatic Lipid Deposition by Activating Ampk Pathway and Beclin1 Phosphorylation Levels to Activate Lipophagy in Tilapia *Oreochromis niloticus*

**DOI:** 10.3389/fnut.2022.841187

**Published:** 2022-03-17

**Authors:** Xiangyuan Liu, Tao Zhao, Xiaolei Wei, Dianguang Zhang, Wuhong Lv, Zhi Luo

**Affiliations:** Laboratory of Molecular Nutrition for Aquatic Economic Animals, Fishery College, Huazhong Agricultural University, Wuhan, China

**Keywords:** phosphorus, lipid metabolism, AMPK/Beclin1 pathway, phosphorylation, vertebrates

## Abstract

High-phosphorus diet (HPD) reduces lipid deposition and significantly influences lipid metabolism. However, the relevant mechanism is unknown. Herein, using widely-cultured teleost tilapia *Oreochromis niloticus* as the experimental animals, we found that HPD and Pi incubation reduced triglyceride (TG) content (*P* ≤ 0.05), suppressed lipogenesis, activated AMP-activated protein kinase (AMPK) pathway and autophagy (*P* ≤ 0.05), and increased fatty acid β-oxidation and lipolysis in tilapia liver and hepatocytes (*P* ≤ 0.05). Our further investigation indicated that Pi treatments activated the lipophagy and facilitated mitochondrial fatty acid β-oxidation, and according reduced TG deposition (*P* ≤ 0.05). Mechanistically, phosphorus increased the AMPKα1 phosphorylation level at S496 and Beclin1 phosphorylation at S90, and Beclin1 phosphorylation by AMPKα1 was required for phosphorus-induced lipophagy and lipolysis. Our study revealed a mechanism for Beclin1 regulation and autophagy induction in response to high-phosphorus diet, and provided novel evidences for the link between dietary phosphorus addition and lipolytic metabolism via the AMPK/Beclin1 pathway. Our results also suggested that AMPK should be the potential target for the prevention and control of lipid metabolic disorders. Overall, these results suggested that HPD reduced hepatic lipid deposition by activating AMPK pathway and Beclin1 phosphorylation levels to activate lipophagy, which provided potential targets for the prevention and control of fatty liver in fish.

## Introduction

Phosphate is a necessary microelement and plays many functions in important biological processes, such as bone formation, the biosynthesis of phospholipids and nucleic acids, intracellular signaling and energy metabolism (1–3). Studies demonstrated that a high-phosphate diet suppressed hepatic lipogenesis and increased fat oxidation in the vertebrates (4, 5). The liver is a key organ that plays important roles in lipid metabolism. Non-alcoholic fatty liver disease (NAFLD) is an increasing

metabolic disease worldwide, and hepatic TG accumulation makes the liver susceptible to the mediators of inflammatory cytokines, potentially leading to hepatitis, fibrosis, cirrhosis and finally liver failure (6, 7). At present, the curing methods are limited because the pathological mechanism of NAFLD largely remains unclear. Therefore, it is very important to elucidate the mechanism of high phosphorus dietary affecting lipid metabolism, which will provide innovative insights into crucial mechanisms contributing to the inhibition of the fatty liver occurrence.

Autophagy is an evolutionarily conservative and degradation process essential for the maintenance of the energy levels within the cells (8, 9). More than 40 autophagy-related genes (ATGs) have been identified from yeast to mammalian species (10, 11). These genes participated in autophagic initiation, the phagophore nucleation and elongation, the autophagosome maturation and their fusion with the lysosomes. Studies suggest that autophagy mediates the control of lipid metabolism, which was named as autophagy-specific lipophagy (12). When lipophagy was activated, lipid droplets (LDs) were enveloped by the autophagosomes, fused with the lysosomes to form the autolysosomes, and hydrolyzed by the lysosomal acid lipases to fatty acids (12–14). Thus, lipophagy activation will restrain lipid accumulation (15), and reduce the occurrence of NAFLD (16). However, the mechanism underlying Pi-induced activation of lipophagy is largely unknown.

The deposition and utilization of lipids are critical for the maintenance of energy homeostasis within the cells. AMP-activated protein kinase (AMPK), a serine/threonine protein kinase, is an evolutionarily conserved cellular energy sensor that maintains cellular energy homeostasis (17). AMPK is comprised of a catalytic α-subunit and two regulatory subunits of β and γ (17). A reversible phosphorylation site of conserved threonine residue (Thr172) can activate AMPK (17–19). Woods et al. (20) identified the new AMPK phosphorylation sites at these sies of Thr258 and Ser485 (α1) / Ser491 (α2) of AMPK. Thr172 phosphorylation was enough for the AMPK activation, but Thr258 and Ser485 phosphorylation was not essential for AMPK activity (20). Once activated, AMPK down-regulates fatty acid synthesis (21) and promotes fatty acid oxidation (22). Besides, the activated AMPK can result in the autophagy induction (23, 24). AMPK regulates autophagy by phosphorylating Beclin1 at S91, S94 and T388 in mammals (25, 26). However, the molecular mechanism underlying how AMPK regulates autophagy is still largely unknown.

Tilapia *Oreochromis niloticus* is a critical economic fish all over the world, whose annual yields amount to 4.5 million tons in 2018 (27). However, excessive dietary lipids break the balance of lipolysis and lipogenesis in intensive cultivation and promote excessive lipid deposition in the liver of the fish species (28), which causes NAFLD. The fatty liver disease dramatically reduces the growth rate and disease resistance of tilapia, eventually leading to severe economic losses. Thus, it is crucial to explore the potential targets to reduce hepatic fat deposition and fatty liver disease.

## Materials and Methods

The experimental flow chart could be seen in Supplementary Table 1.

### Experiment I (*in vivo* Studies)

#### Experimental Animal

Juvenile tilapia of uniform size were purchased from a local farm (Wuhan, Hubei Province, China). They were kept in a recyclable system for 2 weeks to acclimate the experimental conditions.

#### Diet Preparation

Three diets were produced with dietary phosphorus addition in the form of NaH_2_PO_4_·2H_2_O at the inclusion levels of 0 (low-phosphorus diet, LPD), 0.53 g/100g (middle-phosphorus diet, MPD) and 1.05 g/100g (high-phosphorus diet, HPD) (Supplementary Table 2). The Pi supplemental levels were determined according to previous study (29, 30). When formulating the diets, dry feedstuffs were ground, and weighed and mixed for 20 min. Next, NaH_2_PO_4_·2H_2_O was added, and they were mixed thoroughly for another 20 min. Then, we added the water and mixed them until a dough was formed. Finally, the dough was passed through the pelletizer with the 2·0-mm die in diameter. The diets were oven-dried until the moisture was about 10%. They were kept in the −20 °C. Final dietary phosphorus levels were measured, and the values were 1.21 g/100g (low-phosphorus diet, LPD), 1.75 g/100g (middle-phosphorus diet, MPD) and 2.66 g/100g (high-phosphorus diet, HPD).

#### Tilapia Rearing and Tissue Collection

After acclimating the experimental conditions, 225 tilapia (8.89 ± 0.01g, mean ± S.E.M) were randomly divided into nine circular fiberglass tanks (90 cm height, 80 cm diameter; 300 L water volume). Each experimental diet was assigned into three tanks randomly. The fish were fed to satiation twice a day (08:00 and 16:00, respectively) for 10 weeks. The experiment was conducted at natural photoperiod (approximately 12 h of light and 12 h of darkness). During the experiment, the parameters of the water quality in the tanks were followed below: water temperature 28.0–30.5°C, dissolved oxygen 6.10–6.52 mg/L, pH 7.05–7.59 and NH_4_-N 0.05–0.08 mg/L. The water temperature was measured with thermometer. Dissolved oxygen was measured with portable DO instrument. pH was measured with portable pH meter, and NH_4_-N with Nessler reagent spectrophotometry (GB/T 7479-1987). After the 10-week experiment, before collecting the samples, tilapia was fasted for 24 h. All sampled tilapia were anesthetized using MS-222 (100 mg/L water). Then, the liver tissues from ten fish were obtained from each tank, immediately frozen in liquid nitrogen and stored at −80°C for the RNA and protein isolation. The liver of another 3 fish was collected in each tank. They were fixed in 2.5% paraformaldehyde and 10% neutral buffered formalin for the histological, histochemical and ultrastructural analysis, respectively. Another 3 fish were collected and their livers were obtained for analysis of TG content and enzyme activities. The livers of another 6 fish were frozen in the liquid nitrogen and stored at −80°C for subsequent analysis.

#### TG, Non-esterified Fatty Acid (NEFA), Phosphorus Contents, and Enzymatic Activities

Commercial kits were used to determine TG (A110-1-1, Nanjing Jiancheng Bioengineering Institute, Nanjing, China, detection limit: 0–9.04 mmol/L) and NEFA contents (A042-1-1, Nanjing Jiancheng Bioengineering Institute, Nanjing, China, detection limit: 0.01–2.0 mmol/L) according to the manufacturer's instructions, respectively. Phosphorus content of diets and the liver was determined using the molybdovanadate method (spectrophotometry) as described previously (31). Fatty acid synthase (FAS) activity was measured according to the method of Chang et al. (32) and Chakrabarty (33), 6-phosphogluconate dehydrogenase (6PGD) and glucose 6-phosphate dehydrogenase (G6PD) were determined by the method of Barroso et al. (34), malic enzyme (ME) by Ochoa [Ochoa (35)], isocitrate dehydrogenase (ICDH) according to the method of Bernt and Bergmeyer (36) and carnitine palmitoyltransferase 1 (CPT1) was analyzed using the method of Bieber and Fiol (37). The amount of enzyme was defined that converts 1 μM of the substrate to product per minute at 30 °C. The one unit of enzyme activity was expressed as units per milligram of soluble protein, and Bradford Protein Assay Kit (W042-1-1, Nanjing Jiancheng Bioengineering Institute, Nanjing, China, detection limit: 20–2000 μg/ml) was used to determine soluble protein concentration.

#### Oil Red O (ORO) and Hematoxylin-Eosin (H&E) Staining, Transmission Electron Microscopy (TEM) Observation

ORO staining was conducted according to Spisni et al. (38). H&E staining was conducted according to Woods and Ellis (39). In total 10 fields from each sample were examined randomly to quantify the relative areas of hepatic lipid droplets in ORO and vacuoles in H&E staining by the Image J software. TEM observation has been described by recent publications (40).

#### Quantitative Real-Time PCR (QPCR) for MRNA Expression Analysis of Genes

The qPCR assays were performed to quantify the mRNA expression of genes, based on these in our previous research (41). In brief, total RNA was isolated using in TRIzol^TM^ reagent (15596018, Invitrogen, USA), and transcribed into the cDNA with reverse transcription kit (4368813, Invitrogen, USA). qPCR assays were carried out in a 10 μL reaction system, 2 × SYBR^®^Premix Ex Taq^TM^ (TaKaRa) 5 μL, 10 mM each of forward and reverse primers 0.2 μL, 0.6 μL diluted cDNA template and 4 μL double distilled H_2_O. The specific primer sequences for the qPCR analysis are listed in Supplementary Table 3. Seven housekeeping genes (*b2m, rpl7, hprt*, β*-actin, ubce, 18s rRNA* and *tuba*) were selected to screen out two stable genes as the endogenous controls, based on the analysis of the geNorm. The methods 2^−Δ*ΔCt*^ was used to calculate the relative mRNA expression of each gene.

#### Western Blot

Western blotting analysis was conducted to analyze the protein expression according to our recent publications (42). In brief, tilapia liver tissues and hepatocytes were washed in the 1 X PBS. They were placed in the RIPA lysis buffer (G3424, GBCBIO Technologies, Guangdong, China) and oscillated in physical and ultrasonic systems, respectively. Then, they were kept on the ice for 30 min and then centrifuged at 12000 rpm at 4°C for 10 min. We used the BCA assay (G3522-2, GBCBIO Technologies, Guangdong, China. The detection limit: 50–2000 μg/ml) to quantify protein. Next, 15% SDS–polyacrylamide gel was used to separate proteins (40 μg), which were transferred to PVDF membranes. Then, the blots were blocked with the 8% (w/v) skimmed milk in TBST for 2 h and washed thrice in the TBST buffer for 5 min each time. They were then incubated overnight with the specific primary antibodies at 4°C. These specific primary antibodies included the rabbit anti-AMPKα1(1:1000, ET1608-40, HUABIO, Hangzhou, China), anti-phospho-AMPKα1(1:500, ET1612-72, HUABIO, Hangzhou, China), anti-Beclin1 (1:2000, A7353, ABclonal, Wuhan, China), anti-phospho-Beclin1 (1:1000, AP1254, ABclonal, Wuhan, China), anti-SQSTM1/p62 (1:500, #5114, Cell Signaling Technology, Boston, USA), anti-LC3B (1:1000, ab51520, Abcam, MA, USA), anti-GAPDH (1:10000, #2118, Cell Signaling Technology, Boston, USA ), anti-GFP tag (1:2000, AE012, ABclonal, Wuhan, China) and 6 × His-tag (1:2000, 66005-1-Ig, Proteintech, Wuhan, China). Next, membranes were incubated with the corresponding secondary antibodies: the HRP-conjugated antirabbit IgG antibody (7074, Cell Signaling Technology, Boston, USA), HRP-294 conjugated mouse antirabbit IgG (5127, Cell Signaling Technology, Boston, USA), or HRP-conjugated antirabbit IgG antibody (light chain-specific) (93702, Cell Signaling Technology, Boston, USA). Finally, the membranes were visualized with the enhanced chemiluminescences and quantified by the Image-Pro Plus 6.0.

### Experiment II (*in vitro* Studies)

#### Tilapia Hepatocytes Culture and Treatments

To explore the mechanism of dietary phosphorus on lipid metabolism, we isolated the primary hepatocytes from the tilapia and cultured them as described previously (43). Approximately 300 male tilapia were from the same original batch as used in Experiment I (8.89 ± 0.01g, mean ± S.E.M). We designed two treatments: the control (without extra Pi addition), 3.0 mmol/L Pi in the form of Na_2_HPO_4_·12H_2_O/NaH_2_PO_4_·2H_2_O. The Pi concentrations were used based on previous studies (44) and our pilot trials, which did not affect cell viability. We designed specific small interfering RNA (siRNA) for knock-downing the *ampk*α*1* and *beclin1* genes to determine the effect of the AMPK/Beclin1 pathway on lipid metabolism in tilapia. Tilapia hepatocytes were incubated with the inhibitor chloroquine (CQ) of the autophagy-lysosomal pathway (CQ; C6628, MilliporeSigma, MA, USA) to investigate whether and how autophagy mediated dietary phosphorus-induced lipolysis. The primary hepatocytes were incubated in the control or Pi group in the L-15 medium for 48 h with or without 2-h pre-incubation with 5μM CQ.

#### The MTT Assay for Cell Viability

We used the 3-(4,5-dimethyl-2-thiazolyl)-2,5-diphenyl-2-H-tetrazolium bromide (MTT) assay to test the cell viability. The protocols were based on the publication (45).

#### Plasmid Construction, SiRNA Interreference and Cell Transfections

To identify the phosphorylation sites of Beclin1 and AMPKα1, we constructed the vectors of Beclin1 and AMPKα1 expression according to our publication (46). The open reading frames (ORFs) of Beclin1 and AMPKα1 sequences were subcloned into the pcDNA3.1 (+) vector with the GFP-tag and the His-tag, respectively. They were named GFP-Beclin1 and His-AMPKα1, respectively. We produced the mutations of AMPKα1 serine (S) 496 to alanine (A) in the His-AMPKα1 plasmid and mutations of Beclin1 serine (S) 90 to alanine (A) in the GFP-Beclin1 plasmid by the Mut Express II Fast Mutagenesis Kit (C112-02, Vazyme, Piscataway, NJ, USA). The transient transfection of the plasmids into the HEK 293T cells was performed using Lipo293^TM^ transfection reagent (C0521, Beyotime, Shanghai, China). For Beclin1 and AMPKα1-knockdown experiment, the qPCR analysis was used to determine the knockdown efficiencies of these siRNA sequences. The siRNA sequences were given in Supplementary Table 3. Cell transfections was operated based on the protocols (46). Cells were treated for 48h to investigate the potential mechanism that AMPK and Beclin1 mediated phosphorus-induced lipolysis and lipophagy.

#### Bodipy 493/503 Staining and Immunostaining

The Bodipy 493/503 staining was performed by our published methods (42). At first, tilapia hepatocytes were stained after Pi treatments, and then, flow cytometry or laser scanning confocal microscopy (Leica) were used to analyze and image, respectively. The microtubule-associated protein 1 light chain 3B (LC3B), Lyso-Tracker Red and LDs were stained to investigate their colocalization. Tilapia hepatocytes were fixed with 4% formaldehyde, and then the cells were blocked for 1 h in 5% BSA. Next, the hepatocytes were incubated with the anti-LC3B at 4°C overnight and then incubated with the goat anti-rabbit IgG H&L secondary antibody. The images were captured with the Leica laser scanning confocal microscope (Germany), and the fluorescence intensity was quantified by the software Image J.

#### Immunoprecipitation and Western Blotting

The immunoprecipitation analysis was performed based on these protocols (47). The hepatocytes were lysed in the NP40 cell lysis buffer (p0013F, Beyotime, Shanghai, China) with phosphorylase inhibitor (P1082, Beyotime, Shanghai, China). Then, the anti-GFP tag or anti-His tag was added to the cell lysate overnight at 4 °C and the protein A/G beads (P2012, Beyotime, Shanghai, China) were added. Finally, the immunocomplexes were washed by NP40 buffer. The western blot was used to measure the protein expression.

### Statistical Analysis

The data were presented as mean ± S.E.M. The Prism 8 software (GraphPad Software, CA, USA) was used to analyze the data. For two groups, Student's *t* tests (unpaired, two-tailed) were performed. The data among the three groups were evaluated by one-way analysis of variance and then by the *post-hoc* Duncan's multiple range test to determine the statistical significance. For four groups, the homogeneity of the variances was analyzed by the Levene's test, and one-way ANOVA determined statistical significance with the Bonferroni *post-hoc* test. *P* ≤ 0.05 was considered statistically significant.

## Results

### *In vivo* Studies

#### Growth Performance and Feed Utilization

The survival was not influenced significantly by dietary phosphorus (Supplementary Table 4). Weight gain (WG), specific growth rate (SGR), condition factor (CF) and feed intake (FI) increased, but feed conversion rate (FCR) reduced with increasing dietary phosphorus levels (*P* ≤ 0.05). Hepatosomatic index (HSI) showed no significant differences among the three treatments.

#### HPD Reduced TG Content, Suppressed Lipogenesis and Activated Lipolysis

**Figure 1:**
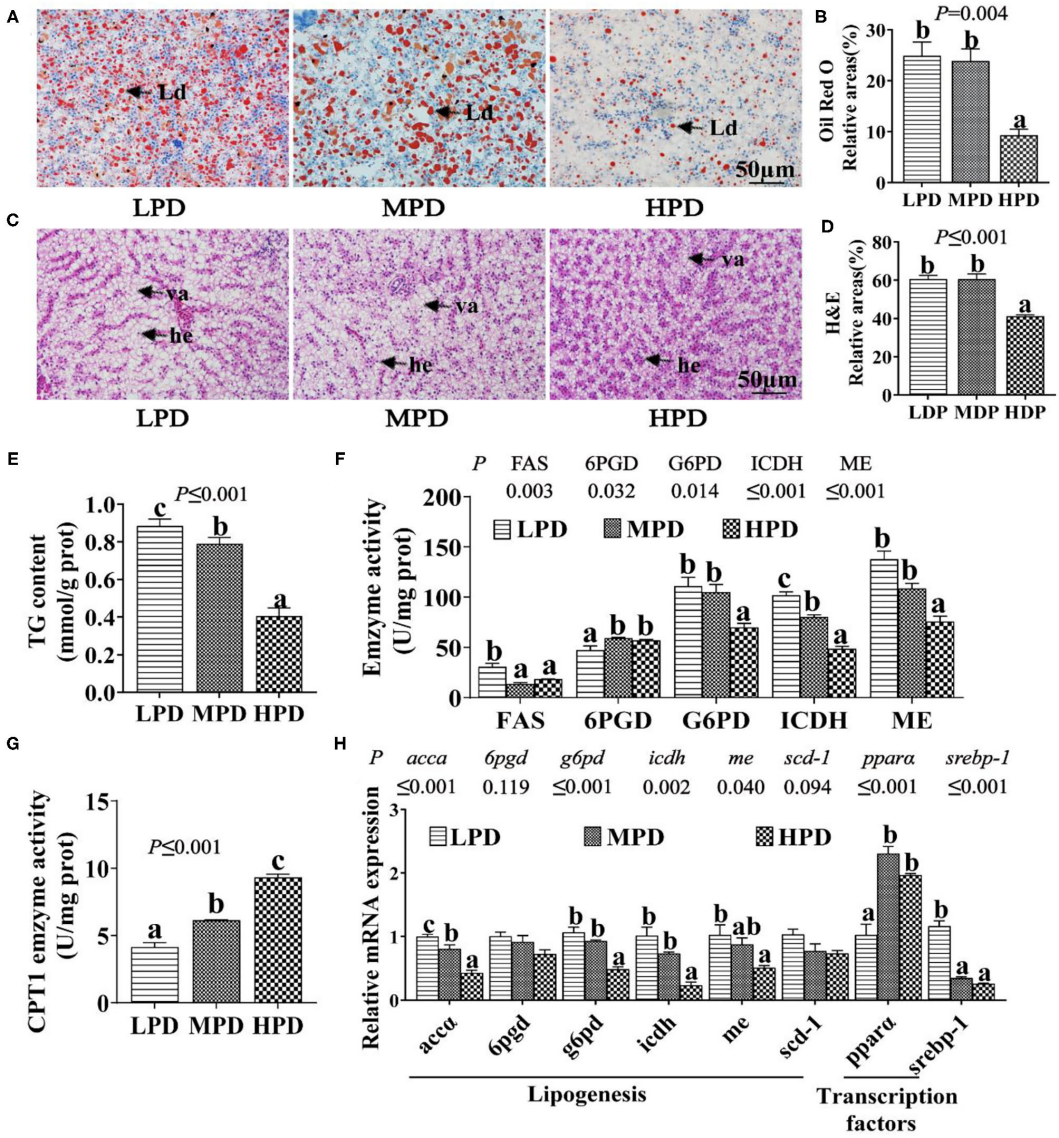
Dietary phosphorus supplementation induced lipid degradation in tilapia liver. **(A)** Representative ORO staining images. Scale bar, 50 μm. **(B)** Quantitative areas for lipid droplets after the ORO staining. **(C)** Representative hepatic H&E staining images. Scale bar, 50 μm. **(D)** Quantitative areas for hepatic vacuoles after the HandE staining. Ld, lipid droplet; he, hepatocyte; va, vacuole. **(E)** TG content. **(F)** Activities of lipogenic enzymes. **(G)** CPT I activity. **(H)** Relative mRNA levels of genes related to lipid metabolism. Data are mean ± SEM (*n* = 3). “a-c” denote significance at *P* ≤ 0.05. The *P* value was calculated by the one-way ANOVA and the *post-hoc* Duncan's multiple range test.

The total hepatic phosphorus content increased with dietary phosphorus levels (*P* ≤ 0.05) (Supplementary Figure 1). Compared to the LPD group, the HPD group tended to reduce hepatic TG content, as shown in ORO and H&E staining, and analysis of TG content (*P* ≤ 0.05) (Figures 1A–E). The HPD group also reduced the activities of lipogenic enzymes (FAS, G6PD, ICDH and ME) (*P* ≤ 0.05), but increased the activity of lipolytic enzyme CPT1 (*P* ≤ 0.05); the HPD group also reduced the mRNA expression of lipogenic genes (*acc*α*, g6pd, icdh, me* and *srebp-1*) (*P* ≤ 0.05), but increased the mRNA expression of the lipolytic gene *ppar*α (peroxisome proliferator-activated receptor α) (*P* ≤ 0.05) (Figures 1F,G). These results indicated that dietary phosphorus addition reduced TG content, suppressed lipogenesis and activated lipolysis in the liver of tilapia.

#### HPD Promoted Autophagy Activity and Increased Fatty Acid (FA) β-Oxidation

**Figure 2:**
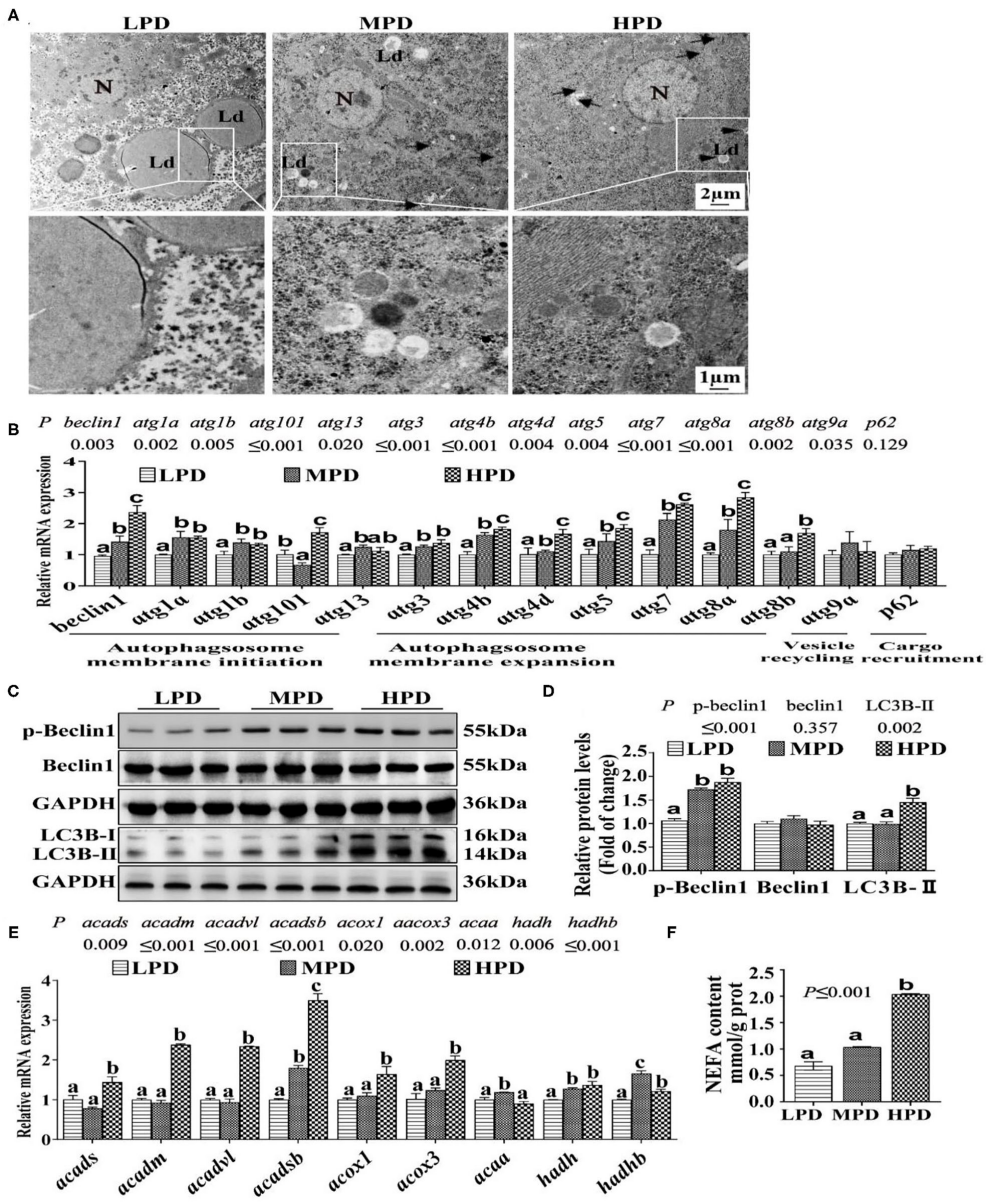
Dietary phosphorus addition increased autophagosome formation in tilapia liver. **(A)** Representative image of liver ultrastructure (TEM). N, hepatocyte nucleus, Ld, lipid droplet. Arrows indicate the autophagosomes and autolysosomes. Arrowheads indicate the lipophagy. **(B)** Relative mRNA levels of genes related to autophagy. **(C,D)** Western blot analysis of Beclin1, p-Beclin1 and LC3B protein levels. **(E)** mRNA levels of the FA β-oxidation-related genes. **(F)** NEFA content. Data are mean ± SEM (*n* = 3). “a-c” denote significance at *P* ≤ 0.05. The *P* Value was calculated by the one-way ANOVA and further the *post-hoc* Duncan's multiple range test.

Since the study pointed out that autophagy regulates lipid metabolism (12), we determine effects of dietary phosphorus addition on the autophagic activity. Compared to the LPD group, HPD group increased autophagosome formation and reduced the number of LDs (Figure 2A). Compared to the LPD group, HPD group up-regulated the mRNA levels of *beclin1, atg1a, atg1b, atg101, atg13, atg3, atg4b, atg4d, atg5, atg7, atg8a* and *atg8b* (*P* ≤ 0.05) (Figure 2B), increased the protein levels of LC3B-II and the phosphorylation level of Beclin1 (*P* ≤ 0.05) (Figures 2C,D), and up-regulated the mRNA expression of many fatty acid β-oxidation genes (*acads, acadm, acadvl, acadsb, acox1, acox3, hadh* and *hadhb*) (*P* ≤ 0.05) (Figure 2E), and increased NEFA content (*P* ≤ 0.05) (Figure 2F). Thus, these results demonstrate that HPD enhanced autophagic activity and increased fatty acid β-oxidation.

#### HPD Activated AMPK Pathway

**Figure 3:**
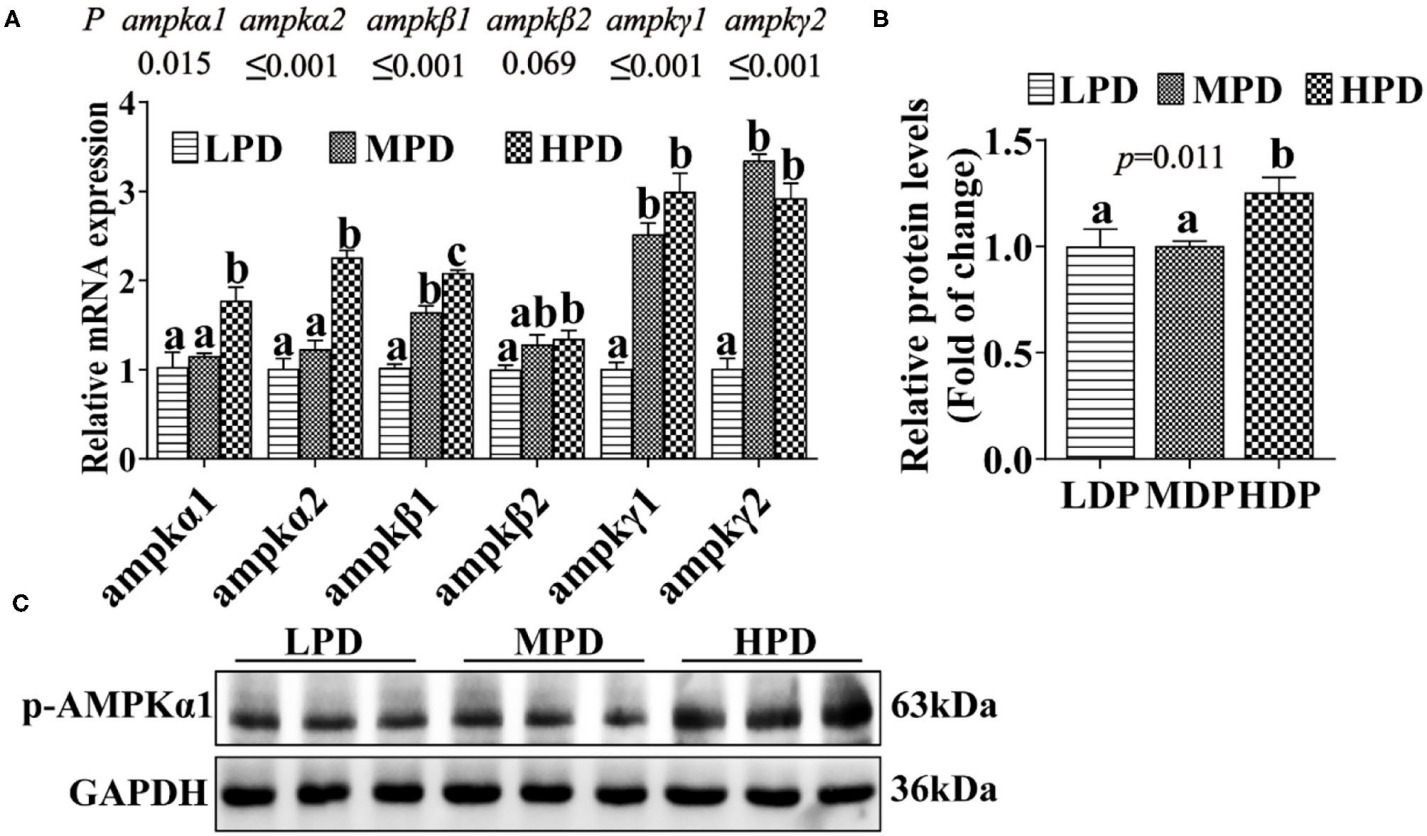
Dietary phosphorus supplementation activated the AMPK pathway and increased AMPKα1 phosphorylation level in tilapia liver. **(A)** Relative mRNA levels of genes related to AMPK pathway. **(B,C)** Western blot analysis of p-AMPKα1 protein levels. Data are mean ± SEM (*n* = 3). “a–c” denote significance at *P* ≤ 0.05. The *P* value was calculated by the one-way ANOVA and further the *post-hoc* Duncan's multiple range testing.

To assess the effect of HPD on AMPK pathway, we analyzed the expression of the key genes and proteins related to the AMPK pathway (Figure 3A). Compared to the LPD group, the HPD group increased mRNA levels of the *ampk*α*1, ampk*α*2, ampk*β*1, ampk*β*2, ampk*γ*1* and *ampk*γ*2* (*P* ≤ 0.05) (Figure 3A), and increased the AMPKα1 phosphorylation level (*P* ≤ 0.05) (Figures 3B,C). These indicated that dietary phosphorus addition activated AMPK pathway in the liver of tilapia.

### *In vitro* Studies

#### Pi Promoted Lipid Degradation

To elucidate the mechanisms of Pi influencing lipid metabolism of tilapia, tilapia hepatocytes were isolated. The MTT assay showed that Pi concentration of 0–3 mM did not adversely influence hepatocyte viability (*P* ≤ 0.05) (Supplementary Figure 2A). Thus, we chose 3 mM for our study. Compared with the control, Pi treatment significantly reduced TG content of hepatocytes, as shown by the analysis of TG contents, and by the flow cytometric analysis and the confocal microscopy after Bodipy 493/503 staining (*P* ≤ 0.05) (Supplementary Figures 2B–E). These data suggested that Pi incubation promoted lipid degradation of tilapia hepatocytes.

#### Pi Activated AMPK Pathway and Enhanced AMPK Phosphorylation Levels

**Figure 4:**
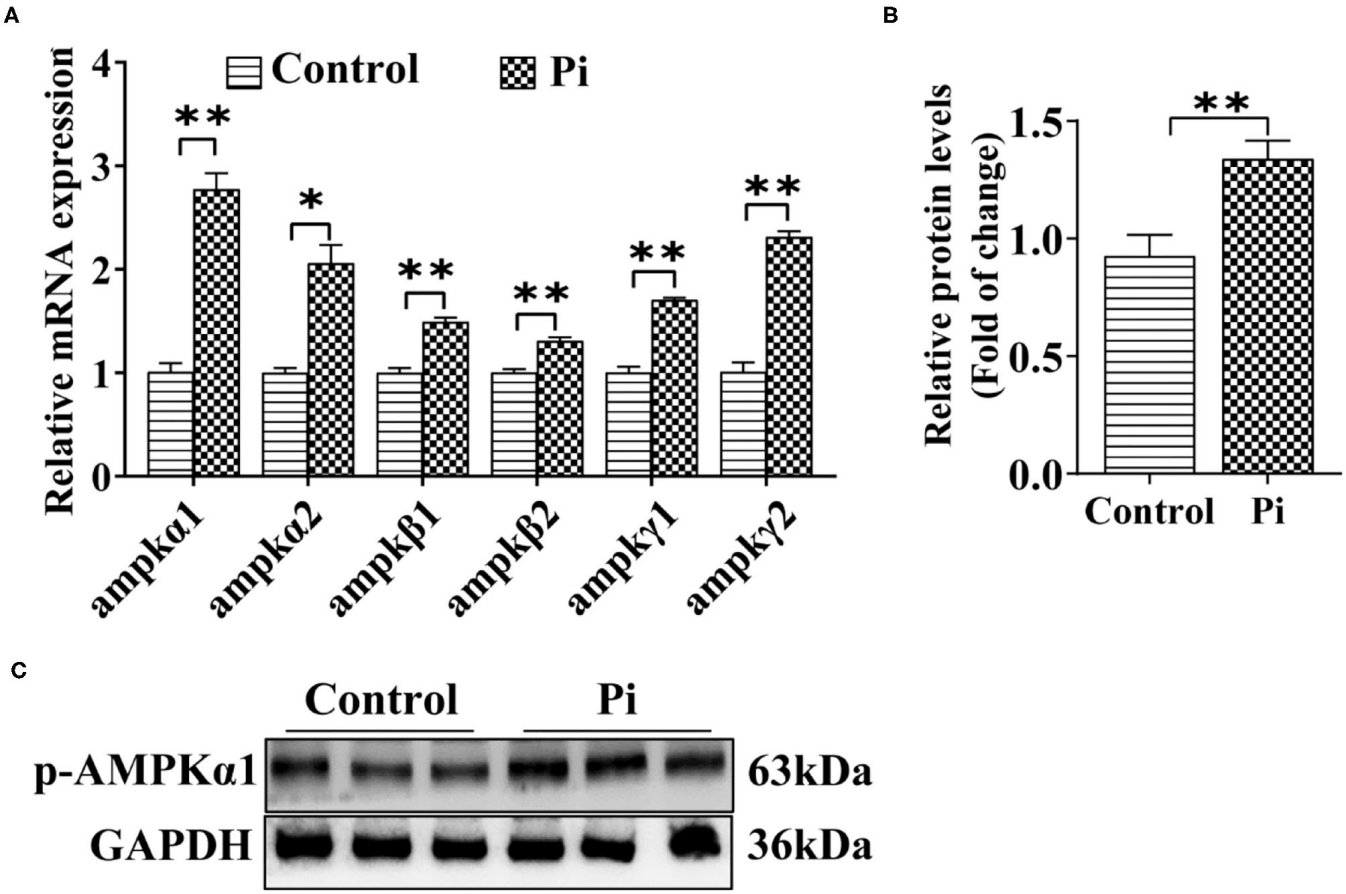
Phosphorus incubation activated AMPK pathway in tilapia hepatocytes. **(A)** Relative mRNA abundances of genes related to AMPK pathway. **(B,C)** Western blot analysis and quantification analysis of p-AMPKα1 protein levels. All data are expressed as mean ± S.E.M (*n* = 3). *P* value was calculated by the Student's *t* tests. *P ≤ 0.05, **P ≤ 0.01, compared with the control.

To determine whether Pi activated the AMPK pathway and accordingly induced autophagy, the effect of Pi on the AMPK pathway in tilapia hepatocytes was investigated. Compared to the control, Pi treatment up-regulated mRNA abundances of *ampk*α*1, ampk*α*2, ampk*β*1, ampk*β*2, ampk*γ*1* and *ampk*γ*2* (*P* ≤ 0.05) (Figure 4A), and increased the p-AMPKα1 protein levels (*P* ≤ 0.05) (Figures 4B,C). These indicated that Pi activated AMPK pathway and enhanced AMPK phosphorylation levels in tilapia hepatocytes.

#### Pi Promoted Lipophagy to Degrade LDs

**Figure 5:**
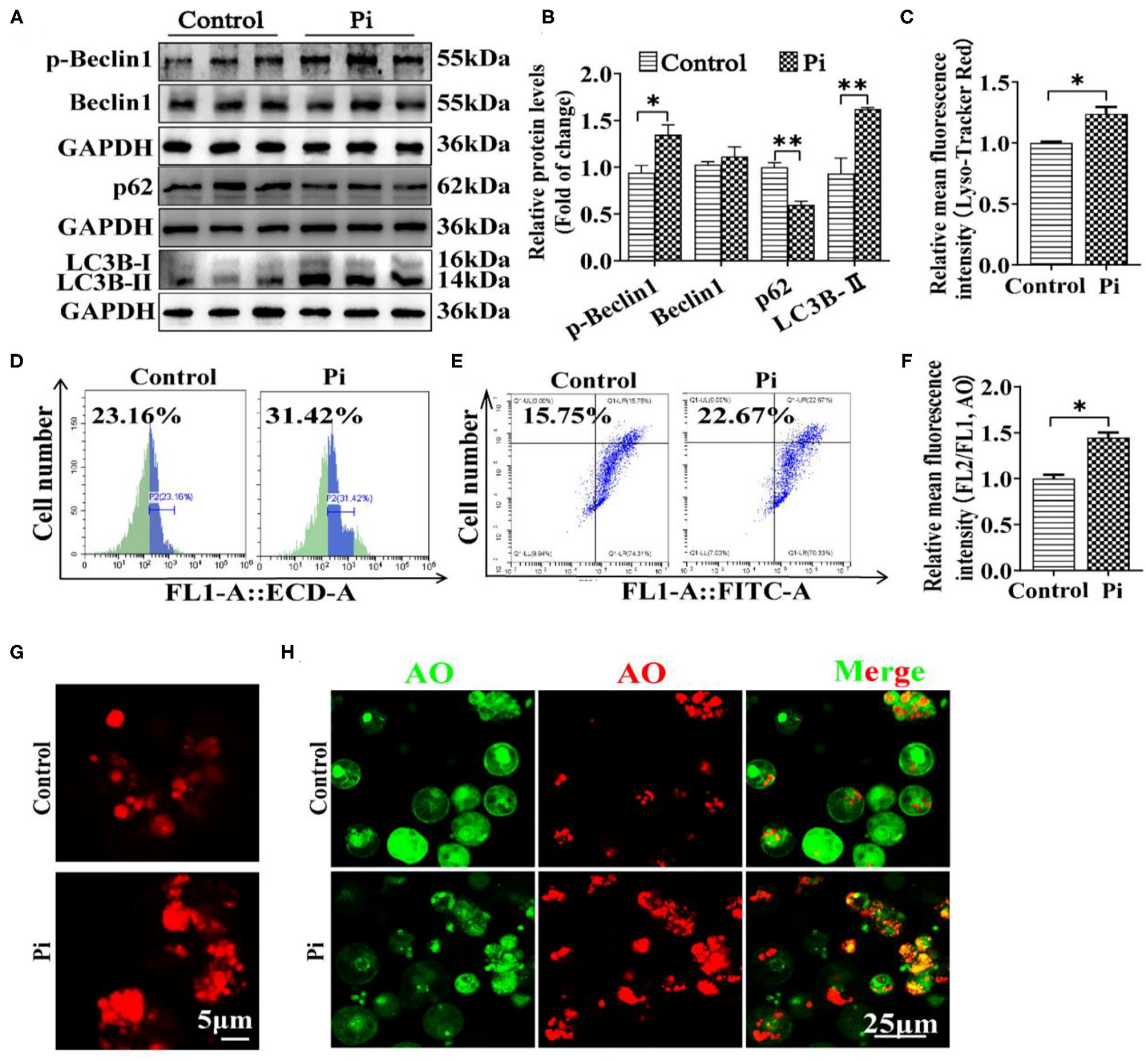
Phosphorus incubation activated autophagy in tilapia hepatocytes. **(A,B)** Western blot analysis and quantification analysis of Beclin1, p-Beclin1, p62 and LC3B protein levels. **(C)** The Lyso-Tracker Red (50 nM)-stained autolysosome was calculated by the flow cytometric analysis of the mean fluorescence intensity. **(D)** The presence of Lyso-Tracker Red-stained autolysosome was demonstrated by the flow cytometry analysis of the mean fluorescence intensity. **(E)** The presence of AO-stained autophagic vacuole was demonstrated by the flow cytometry analysis of the mean fluorescence ratio. **(F)** The AO (1 μM)-stained autophagic vacuoles were calculated by the flow cytometric analysis of red/green (FL2/FL1) fluorescence ratio. **(G)** Lyso-Tracker Red (50 nM)-stained. Scale bar, 5 μm. **(H)** Representative confocal microscopic images of hepatocytes stained with acridine orange. Scale bar, 25 μm. All data are expressed as mean ± S.E.M (*n* = 3). *P* value was calculated by the Student's *t* tests. *P ≤ 0.05, **P ≤ 0.01, compared with control.

In order to confirm whether Pi incubation activated autophagy, we determined the protein levels of several autophogic markers (Beclin1, p-Beclin1, LC3B-II and p62). Compared to the control, Pi incubation increased the protein level of the p-Beclin1 and LC3B-II, and reduced the p62 expression (*P* ≤ 0.05) (Figures 5A,B). Lyso-Tracker and acridine orange (AO) staining demonstrated that Pi increased the autolysosomal formation (red dots), indicating that Pi incubation activated autophagic flux (*P* ≤ 0.05) (Figures 5C–H).

**Figure 6:**
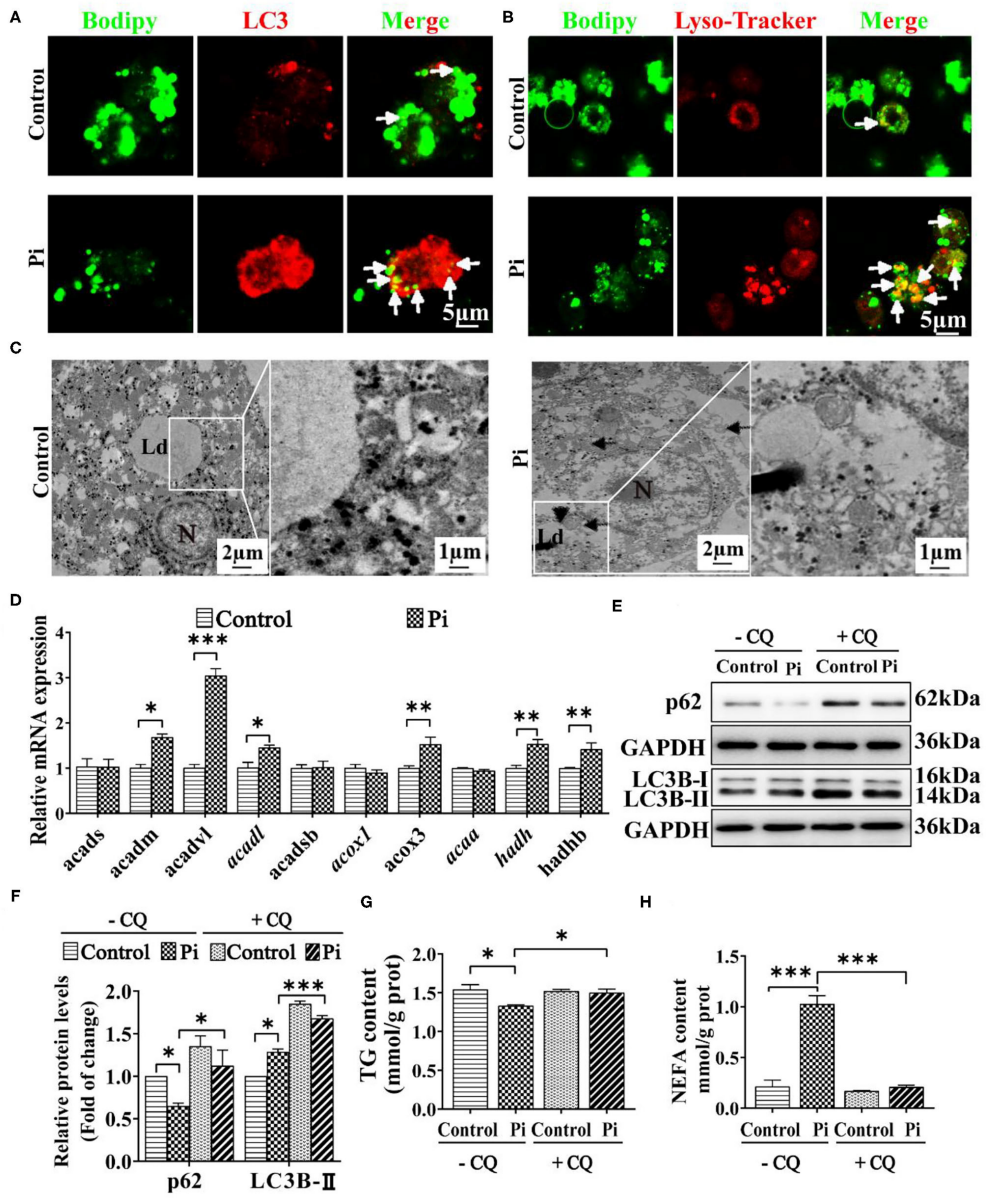
Phosphorus incubation promoted lipophagy in tilapia hepatocytes. **(A)** Confocal microscopic images of the hepatocytes stained with Bodipy 493/503 and LC3 protein. Arrows indicate the colocalization of LC3 protein with LDs. Scale bar, 5 μm. **(B)** Confocal micrograph of hepatocytes stained with Bodipy 493/503 and autolysosome. Arrows indicate autolysosome puncta colocalized with LDs. Scale bar, 5 μm. **(C)** Representative TEM images of hepatocytes after the control or Pi incubation. N, hepatocyte nucleus, Ld, lipid droplet. Arrows indicate the autophagosomes and autolysosomes. Arrowheads indicate the lipophagy. **(D)** Relative mRNA levels of the FA β-oxidation-related genes. **(E, F)** Western blot analysis and quantification analysis of p62 and LC3B protein levels with or without CQ pretreatment (5 mM CQ). **(G)** TG content with or without CQ pretreatment (5 mM CQ). **(H)** NEFA content with or without CQ pretreatment (5 mM CQ). Data are mean ± SEM (*n* = 3), for four groups, *P ≤ 0.05, **P ≤ 0.01, ***P ≤ 0.001, as determined by one-way ANOVA with the Bonferroni *post-hoc* test. For two groups, *P* value was calculated by the Student's *t* tests. *P ≤ 0.05, **P ≤ 0.01, ***P ≤ 0.001, compared with the control.

To confirm whether phosphorus-induced autophagy was linked with the LD degradation, the double immunofluorescences were conducted. The LC3 puncta were linked with the autophagosome formation (48), and their colocalization demonstrated a direct association between LDs and autophagosomes (12). Herein, Pi incubation increased the amounts of LDs (green) bound to LC3 puncta (red) and autolysomes (red) (Figures 6A,B), indicating that phosphorus promoted the autophagosome formation, which helps sequester LDs for degradation. TEM observation confirmed that the LDs integrated with the autolysosomes after Pi incubation, indicating that Pi induced lipophagy (Figure 6C). Singh et al. (12) pointed out that the lipophagy promoted the lipolysis and FA β-oxidation. Accordingly, we tested mRNA expression of genes relevant to FA β-oxidation. Compared to the control, Pi incubation increased the mRNA levels of fatty acid β-oxidation genes (*acadm, acadvl, acadl, acox3, hadh* and *hadhb*) (*P* ≤ 0.05) (Figure 6D). Thus, our data suggested that Pi treatments activated the lipophagy and facilitated mitochondrial fatty acid β-oxidation, and according reduced TG deposition.

To address potential roles of autophagy in Pi-induced lipid degradation and metabolism, we first used CQ, a pharmacological inhibitor of autophagy. CQ pre-incubation alleviated the Pi-induced decrease of p62 protein expression and aggravated the Pi-induced increment of LC3B-II protein expression (*P* ≤ 0.05) (Figures 6E,F). Meanwhile, CQ pretreatment alleviated the Pi-induced decrease of TG content (*P* ≤ 0.05) (Figure 6G), and attenuated Pi-induced increment of NEFA content (*P* ≤ 0.05) (Figure 6H). Taken together, these results proved that lipophagy mediated the Pi-induced lipid degradation in tilapia hepatocytes, and thus showed a causal link between phosphorus-induced lipophagy and LD degradation.

#### Beclin1 Was Required for Pi-Induced Lipophagy and Lipolysis

**Figure 7:**
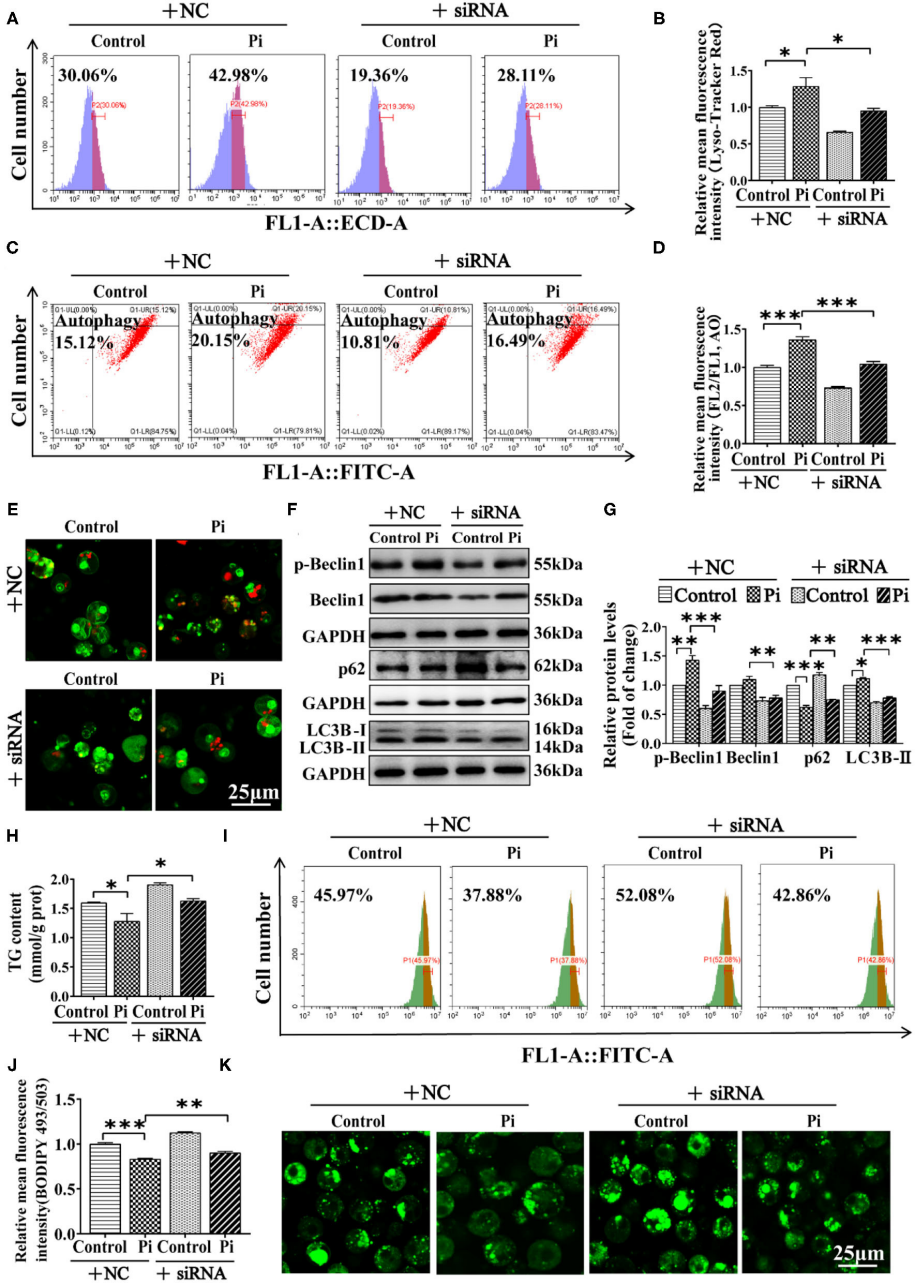
Phosphorus incubation activated lipophagy by triggering Beclin1 phosphorylation to induce lipid degradation in tilapia hepatocytes. Tilapia hepatocytes were incubated with Pi and Beclin1-knockdown experiment for 48h in L-15 medium. **(A)** The presence of Lyso-Tracker Red-stained autolysosome was demonstrated by the flow cytometry, showing the mean fluorescence intensity. **(B)** The Lyso-Tracker Red (50 nM)-stained autolysosome was calculated by the flow cytometric analysis of the mean fluorescence intensity. **(C)** The presence of AO-stained autophagic vacuole was demonstrated by the flow cytometry analysis of the mean fluorescence ratio. **(D)** The AO (1 μM)-stained autophagic vacuole was calculated by the flow cytometric analysis of the mean fluorescence ratio. **(E)** Representative confocal microscopy image of tilapia hepatocytes stained with acridine orange. **(F, G)** Western blot analysis and quantification analysis of the Beclin1, p-Beclin1, p62 and LC3B protein levels. **(H)** TG content. **(I)** The presence of the LDs with Bodipy 493/503 staining was demonstrated by the flow cytometry of tilapia hepatocytes. **(J)** The quantification of Bodipy 493/503-stained LDs by the flow cytometric analysis of FL1 (green) mean fluorescence intensity of tilapia hepatocytes. **(K)** Representative confocal microscopy image of tilapia hepatocytes with Bodipy 493/503 staining. Scale bar, 25 μm. All data are mean ± SEM (*n* = 3), *P ≤ 0.05, **P ≤ 0.01, ***P ≤ 0.001, the *P* Value was calculated by the one-way ANOVA with the Bonferroni *post-hoc* test.

We investigated the possibility of whether Beclin1 was required for Pi-induced lipophagy by knockdown experiment. The siRNA-774 was chosen from three sequences because of its capacity for inhibiting *beclin1* expression (*P* ≤ 0.05) (Supplementary Figure 3). Compared to the control, flow cytometric analysis after LysoTracker and AO staining demonstrated that Beclin1-knockdown alleviated the Pi-induced increment of their fluorescence density (*P* ≤ 0.05) (Figures 7A–D). The confocal microscopic analysis indicated that Beclin1 knockdown alleviated the Pi-induced activation of autophagy (Figure 7E). Moreover, compared to the control, Beclin1 knockdown alleviated the Pi-induced increase of the LC3B-II and p-Beclin1 protein expression, and alleviated the Pi-induced decline of the p62 protein expression (*P* ≤ 0.05) (Figures 7F,G). Meantime, compared to the control, Beclin1 knockdown alleviated the Pi-induced reduction of TG content (*P* ≤ 0.05) (Figure 7H), which was confirmed by the flow cytometry and confocal microscopy after the Bodipy 493/503 staining (*P* ≤ 0.05) (Figures 7I–K). These findings indicated that Beclin1 was required for Pi-induced lipophagy and lipolysis.

#### AMPK Was Required for Beclin1 Phosphorylation in Pi-Induced Lipophagy

**Figure 8:**
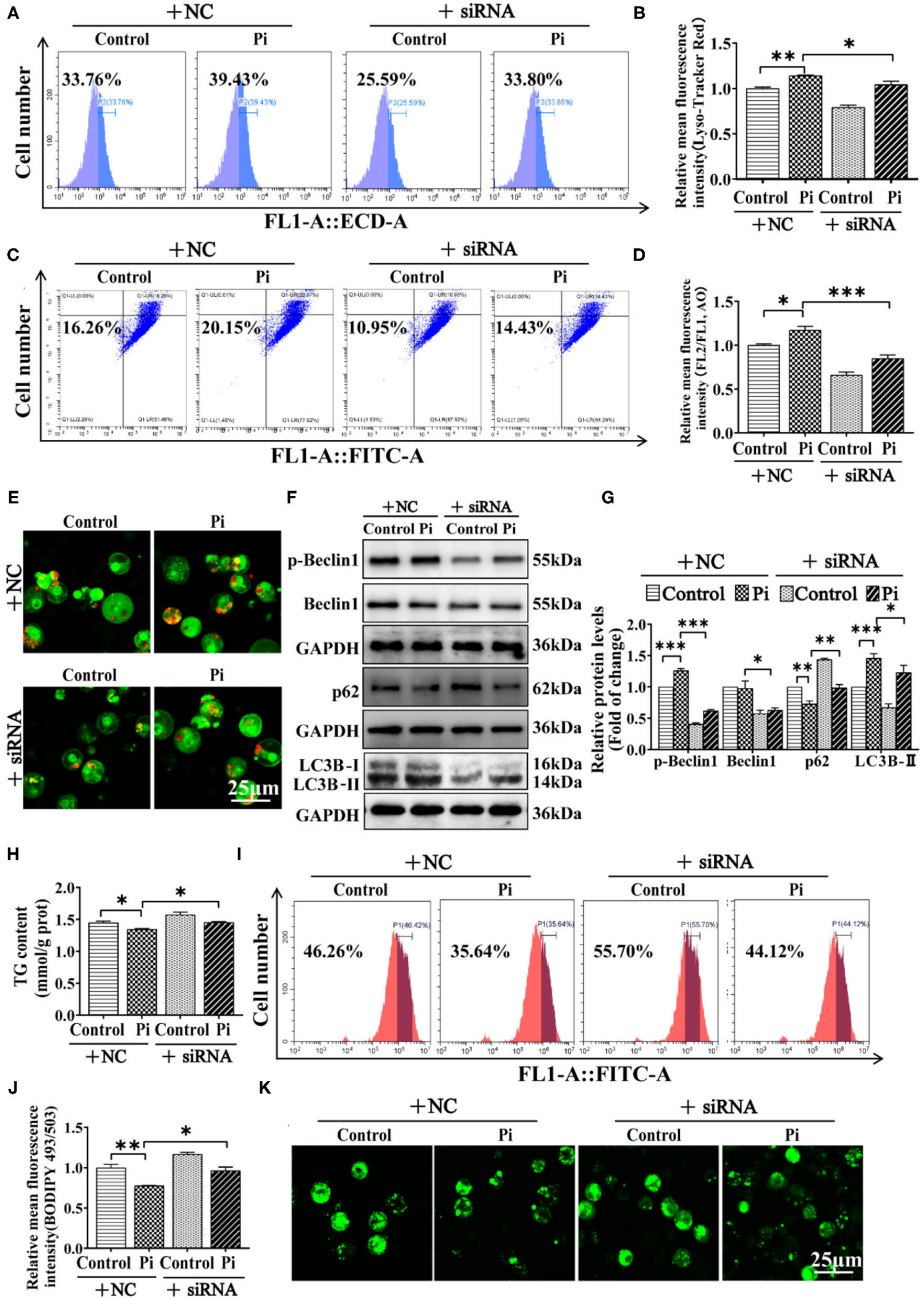
AMPK is required for Beclin1 phosphorylation in phosphorus-induced lipophagy in tilapia hepatocytes. Tilapia hepatocytes were incubated with Pi and AMPKα1-knockdown experiment for 48 h in L-15 medium. **(A)** The presence of Lyso-Tracker Red-stained autolysosomes was demonstrated by the flow cytometry, showing the mean fluorescence intensity. **(B)** The Lyso-Tracker Red (50 nM)-stained autolysosome was calculated by the flow cytometric analysis of the mean fluorescence intensity. **(C)** The presence of AO-stained autophagic vacuole was demonstrated by the flow cytometry analysis of the mean fluorescence ratio. **(D)** The AO (1 μM)-stained autophagic vacuole was calculated by the flow cytometric analysis of the mean fluorescence ratio. **(E)** Representative confocal microscopy image of hepatocytes stained with acridine orange. **(F, G)** Western blot analysis and quantification analysis of the Beclin1, p-Beclin1, p62 and LC3B protein levels. **(H)** TG content. **(I)** The presence of LDs with Bodipy 493/503 staining was demonstrated by the flow cytometry of tilapia hepatocytes. **(J)** Bodipy 493/503-stained LDs was quantified by the flow cytometric analysis of FL1 (green) mean fluorescence intensity of tilapia hepatocytes. **(K)** Representative confocal microscopy image of tilapia hepatocytes with Bodipy 493/503 staining. All data are mean ± SEM (*n* = 3), *P ≤ 0.05, **P ≤ 0.01, ***P ≤ 0.001, the *P* Value was calculated by the one-way ANOVA with the Bonferroni *post-hoc* test.

To establish a direct link between AMPKα1 and Beclin1 in lipophagy, genetic inhibition of *ampk*α*1* by siRNA was used. The siRNA-1374 was chosen from three sequences because of its capability for inhibiting the *ampk*α*1* mRNA expression (*P* ≤ 0.05) (Supplementary Figure 4). Compared to the control, the *ampk*α*1* knockdown inhibited AMPKα1 protein expression (*P* ≤ 0.05) (Supplementary Figures 5A,B). Moreover, flow cytometric analysis and confocal microscopy after Lyso-Tracker and AO staining demonstrated that AMPKα1 knockdown alleviated the Pi-induced increase of autophagic flux (*P* ≤ 0.05) (Figures 8A–E). AMPKα1 knockdown also alleviated Pi-induced increment of the protein expression of the LC3B-II and p-Beclin1, and alleviated the Pi-induced reduction of p62 protein expression. Compared to the control, AMPKα1 knockdown significantly reduced Beclin1 protein expression (*P* ≤ 0.05) (Figures 8F,G). AMPKα1 knockdown also alleviated the Pi-induced reduction of TG content (*P* ≤ 0.05) (Figure 8H). The results were further proved by the flow cytometric analysis and the confocal microscopic observation after Bodipy 493/503 staining (*P* ≤ 0.05) (Figures 8I–K). These indicated that AMPKα1 was required for Beclin1 phosphorylation in lipophagy.

**Figure 9:**
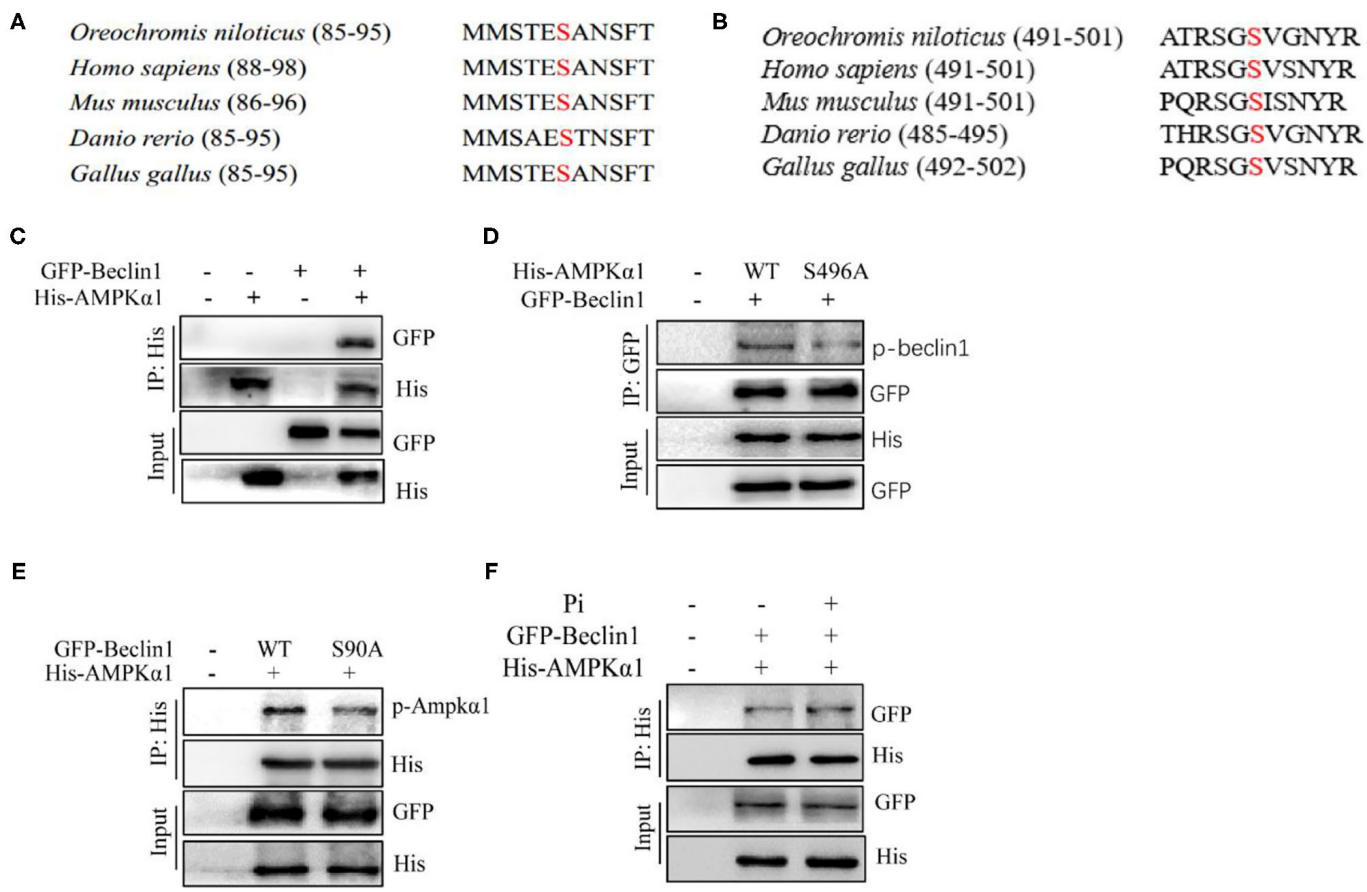
Phosphorylation of Beclin1 by AMPK is necessary for Pi-induced autophagy to degrade LD. **(A)** Alignment of the partial Beclin1 protein sequences among several species. Red S indicates putative serine sites where Beclin1 could be phosphorylated. **(B)** Alignment of partial AMPKα1 protein sequences among several species. Red S indicates putative serine sites where AMPKα1 could be phosphorylated. **(C)** The interaction between AMPKα1 and Beclin1. His-tag AMPKα1 and GFP-tag Beclin1 were transfected into the HEK293T cells. **(D)** Mutations of S496A reduced the Beclin1 phosphorylation level. Phosphorylation of the ectopically expressed WT and S496A was analyzed. **(E)** Mutations of S90A decreased the AMPKα1 phosphorylation level. Phosphorylation of the ectopically expressed WT and S90A was analyzed. **(F)** The interaction between AMPKα1 and Beclin1 after Beclin1 and AMPKα1-overexpression and Pi treatment.

Since HPD increased phosphorylation levels of Beclin1 and AMPKα1, further we investigated the interaction between the two proteins. We aligned the protein sequences of AMPKα1 and Beclin1 among the species, and found that Beclin1 serine 90 and AMPKα1 serine 496 were conserved evolutionarily from fish to mammals (Figures 9A,B). The overexpression plasmid of the tilapia *beclin1* and *ampk*α*1* genes were constructed and transfected into HEK 293T cells to investigate their interaction. We found that AMPKα1 coprecipitated with Beclin1 (Figure 9C). To further validate these findings, site mutations were introduced into the *ampk*α*1* and *beclin1* genes. The S496A mutants of AMPKα1 reduced the Beclin1 phosphorylation level, indicating that tilapia Beclin1 protein could be phosphorylated at the S90 site. Similarly, the S90A mutants of Beclin1 reduced the phosphorylation level of AMPKα1 (Figures 9D,E). Compared to the control, Pi incubation increased the coprecipitation between the Beclin1 and AMPKα1 (Figure 9F). Therefore, these results indicated that Beclin1 could be phosphorylated at the site of S90 by AMPKα1, which supported the hypothesis that AMPKα1-mediated Beclin1 phosphorylation activated lipophagy and promoted lipid degradation.

**Figure 10:**
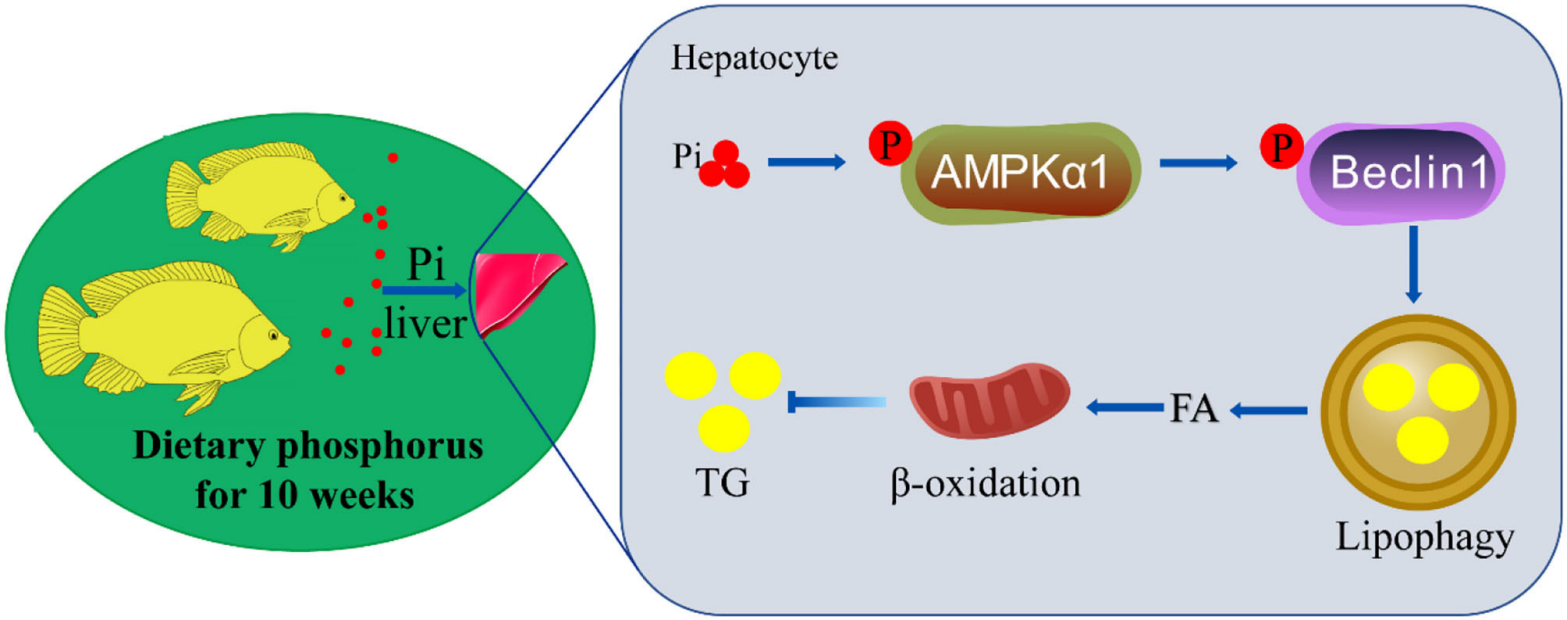
The mechanisms of dietary phosphorus inducing lipid degradation and lipophagy through AMPK/Beclin1 pathways. Dietary phosphorus activated AMPKα1 phosphorylation, triggered lipophagy by phosphorylating Beclin1, and induced lipid degradation of the liver tissues of tilapia.

In summary, we identified an innovative mechanism of coordinated hepatic lipid metabolism mediated by phosphorus-induced lipophagy (Figure 10). HPD activated AMPKα1 via phosphorylating serine residue 496. The activated AMPK phosphorylated beclin1 on Ser90, and led to lipophagy and mitochondrial fatty acid β-oxidation, which promoted lipolysis and reduced lipid deposition.

## Discussion

In our study, total phosphorus content in the liver increased with dietary phosphorus levels, fish fed the high phosphorus dietary increased growth performance and reduced FCR, in agreement with other studies (49). Moreover, compared to the LPD group, the HPD group tended to reduce hepatic TG contents, suppressed lipogenesis and up-regulated lipolysis, similar to other studies (4, 5, 50). Thus, dietary Pi is a novel metabolic regulator, and dietary supplementation of phosphate could be useful for the potential treatment of NAFLD.

Since autophagy regulates lipid metabolism (12, 14), we determined effects of dietary phosphorus on the autophagy. Our study indicated that, compared to the LPD group, HPD group increased autophagosome formation and reduced the number of LDs, up-regulated the mRNA abundances of the *beclin1, atg1a, atg1b, atg3, atg101, atg13, atg4b, atg4d, atg5, atg7, atg8a* and *atg8b*, increased the LC3B-II protein expression and the Beclin1 phosphorylation level. Similarly, Wei et al. (42) found that high Zn diet increased the mRNA levels of *atg1a, atg1b, atg3, atg7 and atg9b*, and up-regulated LC3B-II protein expression. Zhao et al. (14) found that high carbohydrate diets significantly up-regulated mRNA levels of the autophagy-related genes (*atg1a, atg4a, atg5, atg6, atg7, atg8b, atg8a, atg9a* and *atg9b*), and increased the protein expression of autophagy markers (LC3B-II and Beclin1). During the autophagosome formation, the cytosolic LC3-I is conjugated to the phosphatidylethanolamine (PE) to form the LC3-II, which is then bound to the autophagosome (48). Beclin1 is essential for vesicle nucleation and autophagosome formation (8). These indicated that high phosphorus dietary addition induced authophagy, in agreement with the study by Dai et al. (44). Singh et al. (12) pointed out that autophagy degraded the hepatocellular LDs through lipophagy. In the present study, lipid droplets were colocalized within the autolysosomal compartments, and LC3B-II co-localized with lipid droplets. These indicated that phosphorus-induced autophagy was lipid droplet-specific lipophagy. Moreover, Singh et al. (12) found that the increase of lipophagy was accompanied with lipolytic activation and NEFA release. Similarly, our study suggested that high phosphorus dietary addition significantly escalated the mRNA expression of several FA β-oxidation genes (*acads, acadm, acadsb, acadvl, acox1, acox3, hadh* and *hadhb*), and increased NEFA content. Our further investigation suggested that Pi treatments activated the lipophagy and facilitated mitochondrial fatty acid β-oxidation, and according reduced TG deposition. In our *in vitro* study, compared to the control, Pi up-regulated the p-Beclin1 and LC3B-II protein levels, down-regulated p62 expression. Beclin1 phosphorylation is required for the autophagy induction and LC3 accumulation (25). p62 is an important protein that links the ubiquitinated proteins to the autophagic machinery and degrades these proteins in the lysosome (42, 51). The down-regulated p62 expression by Pi indicated the activation of autophagic flux, as suggested by Zhao et al. (14). This mobilization and hydrolysis of TG to NEFA led to increased NEFA delivery to the mitochondria via the lipophagy-dependent pathway, which increased FA β-oxidation. Thus, our results demonstrate that HPD and Pi incubation enhanced autophagic activity, promoted lipophagy and increased FA β-oxidation. In the present study, Beclin1 knockdown alleviated the Pi-induced activation of autophagy, and alleviated the Pi-induced increase of the p-Beclin1 and the LC3B-II protein expression, and alleviated the Pi-induced decline of the p62 protein expression. These further confirm that Beclin1 is key protein for autophagy activation. Moreover, we found that, compared to the control, Beclin1 knockdown alleviated the Pi-induced reduction of TG content. Similarly, Singh et al. (12) found that inhibition of autophagy triggered the increased TG and LD accumulation in hepatocytes. These findings indicated that Beclin1 phosphorylation was required for Pi-induced lipophagy and lipolysis.

AMPK is one of the crucial modulators for lipid homeostasis (52). Herein, compared to the LPD group, the HPD group up-regulated mRNA levels of the *ampk*α*1, ampk*α*2, ampk*β*1, ampk*β*2, ampk*γ*1* and *ampk*γ*2*, and increased the AMPKα1 phosphorylation level. In our *in vitro* study, Pi incubation up-regulated the mRNA abundances of *ampk*α*1, ampk*α*2, ampk*β*1, ampk*β*2, ampk*γ*1* and *ampk*γ*2*, and increased the p-AMPKα1 protein levels. Generally, Pi-induced increase of their expression indicated the activation of AMPK signals. Similarly, Wei et al. (53) indicated that high dietary magnesium addition upregulated mRNA abundances of *ampkb1, ampkb2, ampka1, ampka2, ampkg1a* and *ampkg1b*. To our knowledge, our study is the first report which focused on dietary Pi-induced changes of mRNA concentrations of these genes, indicating that dietary phosphorus addition influenced AMPK pathway. Our further investigation found that AMPKα1 had direct interaction with Beclin1, and Pi incubation increased the coprecipitation of Beclin1 and AMPKα1. Moreover, we found that AMPK was required for Beclin1 phosphorylation in Pi-induced lipophagy. AMPK coordinates the various aspects of the autophagy machinery for induction of autophagosome formation (52). Zhang et al. (26) found that AMPK phosphorylates Beclin1 to induce autophagy. Mechanistically, our study indicated that the tilapia Beclin1 could be phosphorylated at the site of S90 by AMPKα1, indicating that AMPKα1-mediated Beclin1 phosphorylation activated lipophagy and promoted lipid degradation. Beclin1 S91/S94 phosphorylation is particularly important for AMPK-dependent autophagy (25). Our study indicated that the AMPKα1 knockdown alleviated the Pi-induced reduction of TG content. Smith et al. (54) reported that the increased AMPK activity contributed to the inhibition of the fatty liver disease linked with excess lipid production. Our study demonstrated that the activation of AMPK pathway accounted for beneficial influences of phosphorus on lipid metabolism.

## Conclusion

In conclusion, HPD-induced autophagy and lipid turnover involved the activation of AMPK pathway via the AMPKα1 phosphorylation at S496. AMPK promotes phagophore nucleation by phosphorylation Beclin1 at S90. Our data demonstrated that dietary phosphorus supplementation (1.05 g/100g NaH_2_PO4·2H_2_O) can negatively regulate lipid synthesis in the liver, thereby preventing the occurrence of NAFLD, which could provide the potential target for the treatment of NAFLD in practical.

## Data Availability Statement

The raw data supporting the conclusions of this article will be made available by the authors, without undue reservation.

## Ethics Statement

The animal study was reviewed and approved by Huazhong Agriculture University.

## Author Contributions

ZL and XL designed the experiments, analyzed the data, and had primary responsibility for the final content. XL carried out the animal and cell experiments and sample analysis with the help of TZ, XW, DZ, and WL. XL drafted the manuscript and ZL revised the manuscript. All authors read and approved the final manuscript.

## Funding

This study was funded by National Key R&D Program of China (2018YFD0900400).

## Conflict of Interest

The authors declare that the research was conducted in the absence of any commercial or financial relationships that could be construed as a potential conflict of interest.

## Publisher's Note

All claims expressed in this article are solely those of the authors and do not necessarily represent those of their affiliated organizations, or those of the publisher, the editors and the reviewers. Any product that may be evaluated in this article, or claim that may be made by its manufacturer, is not guaranteed or endorsed by the publisher.

## Abbreviations

6PGD: 6-phosphogluconate dehydrogenase
A: alanine
ACC1: acetyl-CoA carboxylase 1
AMPK: AMP-activated protein kinase
AO: acridine orange
ATG: autophagy-related gene
CF: condition factor
CPT1: carnitine palmitoyltransferase 1
CQ: chloroquinec
FA: fatty acid
FAS: fatty acid synthase
FCR: feed conversion rate
FI: feed intake
G6PD: glucose 6-phospate dehydrogenase
GIFT: Genetic Improvement of Farmed Tilapia
H&E: hematoxylin and eosin
HIS: hepatosomatic index
HPD: high-phosphorus diet
ICDH: isocitrate dehydrogenase
LC3B: microtubule associated protein 1 light chain 3B
LD: lipid droplet
LPD: low-phosphorus diet
ME: malic enzyme
MPD: middle-phosphorus diet
NAFLD: non-alcoholic fatty liver disease
NC: negative control
NEFA: non-esterified fatty acid
ORO: oil red O
PPAR: peroxisome proliferator-activated receptor
qPCR: quantitative real-time quantitative PCR
S: serine
SCD-1: stearoyl-CoA desaturase 1
SGR: specifc growth rate
siRNA: short hairpin RNA
SQSTM1/p62: sequestosome 1
SREBP-1: sterol regulatory element-binding proteins-1
TEM: transmission electron microscopy
T: threonine
TG: triglyceride
WG: weight gain.
