# Supplementary material for: Dietary Phosphorus Reduced Hepatic Lipid Deposition by Activating Ampk Pathway and Beclin1 Phosphorylation Levels to Activate Lipophagy in Tilapia Oreochromis niloticus
Source: Front Nutr. 2022 Mar 17;9:841187. doi: 10.3389/fnut.2022.841187 (PMC8969567; doi:10.3389/fnut.2022.841187)
Supplement: Supplementary file 1 [file Data_Sheet_1.docx]

**Supplementary Figure and Table legends**

***Supplementary Table 1*** *Flow chart of the experiment*

***
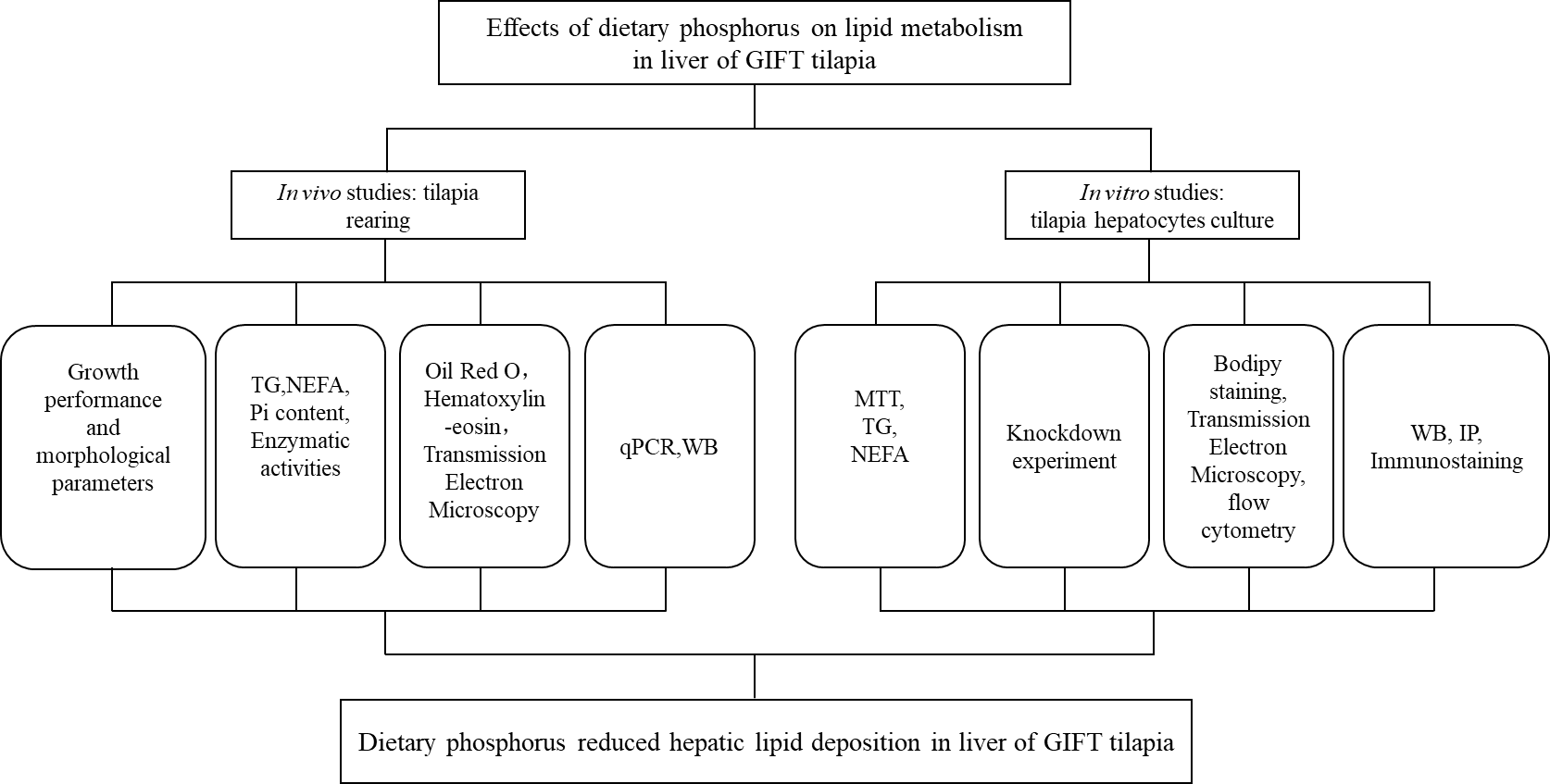
***

***Supplementary Table 2*** *Feed formulation and proximate analysis of experimental diets.*

| Ingredients (g kg^−1^) | LDP | MDP | HDP |
| --- | --- | --- | --- |
| Casein | 300 | 300 | 300 |
| Gelatin | 20 | 20 | 20 |
| Fish meal | 50 | 50 | 50 |
| Soybean oil | 25 | 25 | 25 |
| fish oil | 25 | 25 | 25 |
| Wheat flour | 250 | 250 | 250 |
| NaCl | 2 | 2 | 2 |
| CaCl_2_·2H_2_O | 5 | 5 | 5 |
| Y_2_O_3_ yttrium oxide | 5 | 5 | 5 |
| Vitamin premix * | 10 | 10 | 10 |
| Mineral premix * | 10 | 10 | 10 |
| Betaine | 10 | 10 | 10 |
| Ascorbic acid | 10 | 10 | 10 |
| NaH_2_PO4·2H_2_O | 0 | 5.3 | 10.5 |
| Cellulose | 278 | 272.7 | 267.5 |
| *Proximate analysis (%, dry weight)* |  |  |  |
| Moisture | 9.94 | 10.12 | 9.83 |
| Crude protein | 34.44 | 34.68 | 33.35 |
| Crude Lipid | 6.87 | 6.35 | 7.01 |
| Ash | 3.69 | 4.05 | 4.02 |
| Total phosphorus | 1.21 | 1.75 | 2.66 |

Vitamin premix (mg or IU per kg vitamin premix): vitamin A，1100000 IU；vitamin D_3_，320000 IU；Nicotinic acid，7800mg；vitamin E，2500mg; vitamin B1,1000mg; vitamin B2,2000mg; vitamin B6,1000mg; biotin, 8mg; folic acid,400mg; vitamin B_12_,125mg; vitamin C,18000mg.

Mineral mixture (g/100 g mineral mixture): Calcium chloride，6.44g；calcium lactate, 32.7 g; ferric citrate, 2.97 g；magnesium sulphate，6.27 g；Potassium chloride，25.26 g；sodium chloride，8.60 g；AlCl_3_•6H_2_O, 0.015 g; KI, 0.015 g; CuCl_2_, 0.01 g; MnSO_4_•H_2_O, 0.08 g; CoCl_2_•6H_2_O, 0.1 g; ZnSO_4_•7H_2_O, 0.3 g.

The chemical composition of diets was assessed using the standard AOAC procedures (AOAC, 1995). Briefly, moisture was determined by drying in a drying oven (DHG-9140A; Jinghong Laboratory Instrument Co., Ltd, Shanghai, China) at 105 °C for 4 hours until constant weight. Crude protein content (Nitrogen × 6.25) was determined by the Kjeldahl method after sulfuric acid digestion using an automated Nitrogen Analyzer (SH220, Hannon Instruments Co. Ltd., Jinan, China). Crude lipid was extracted with ether by the method of Soxhlet, and the ash content was detected at 550 °C for 12 h in a muffle furnace (SX2-4-10, Longkou City Electric Furnace Process

Factory, Longkou, China). Phosphorus content of diets was determined using the molybdovanadate method (spectrophotometry) (it is mentioned in the text).

***Supplementary Table 3*** *Primers used for quantitative real-time PCR analysis, plasmid construction and siRNA.*

| **Genes** | Forward primer (5’-3’) | Reverse primer (5’-3’) | Accession no. |
| --- | --- | --- | --- |
| ***fas*** | TCATCCAGCAGTTCACTGGCATT | TGATTAGGTCCACGGCCACA | GU433188 |
| ***6pgd*** | GAAGGGCCTGCTGTTTGTTG | CCCAGTCACAACAAGGCTCT | [XM_003444904.4](https://www.ncbi.nlm.nih.gov/nucleotide/XM_003444904.4?report=genbank&log$=nucltop&blast_rank=1&RID=HW48H8D6013) |
| ***g6pd*** | GAGAAGCCCTTTGGTCGTGA | ATCAAAGTACCCTCCACGGC | XM_005478106 |
| ***icdh*** | TGACAGTTGAGGCAGCAGAG | TGTCTGCCGATGATGATGGG | XM_003449515.4 |
| ***me*** | GAACGCCCCATCATCTTTGC | ACCAGGGTAGAAGGTCCTCC | XM_003453476.5 |
| ***scd-1*** | TCCCCGGATCATCGTATGGA | TCCTGCAGTGACTCCTAACG | XM_005471382 |
| ***pparα*** | CTGATAAAGCTTCGGGCTTCCA | CGCTCACACTTATCATACTCCAGCT | KF871430 |
| ***srebp-1*** | TGCAGCAGAGAGACTGTATCCGA | ACTGCCCTGAATGTGTTCAGACA | XM_005457771 |
| ***becin1*** | ATGTCAACAGAGAGCGCCAA | TGATGTTGAGCTGCGTGTCT | XM_005471281.3 |
| ***atg1a*** | GGACCTCAAGCCACAGAACA | GCCATGTACATAGGGGAGCC | XM_005451336.4 |
| ***atg1b*** | TTGGCTTTCGTCCAGTGTGT | TGGAAACAGCTCGCCTTTCT | XM_005473083.3 |
| ***atg101*** | CCTTGGGAAGTGTGGAGCAT | CTGACTGGGTGGGCATCTTT | XM_025910683.1 |
| ***atg13*** | GTCACCCTGCTTCCAATCCA | TCCGACCTTCCTTGTGAACG | XM_005460641.4 |
| ***atg******4b*** | CCCGATGAGACGTACCACTG | CCATATGAGAGGGCTGGCTG | XM_003444208.4 |
| ***atg4d*** | GATCCTCACTACTGCCAGCC | CCTTCGACGATGAGAGAGCC | XM_003447923.5 |
| ***atg5*** | GACCAGTTCTGGGCCATGAA | GGTGTATGCGTTGCCTACAG | [XM_003450274.5](https://www.ncbi.nlm.nih.gov/nucleotide/XM_003450274.5?report=genbank&log$=nucltop&blast_rank=1&RID=HW0HW4U6016) |
| ***atg7*** | GATGTTGTGGCACCAGGAGA | CATTCTGTCATCGCTGCTGC | XM_025907253.1 |
| ***atg9a*** | TACTGGTTCACGTGCCTGAG | GCTCGATTGGCAAACAGGAC | XM_005476433.4 |
| ***atg8a*** | GTCCAGCAGATCCGTGAGC | TGCCAGGAACTTGGTCTTGTC | XM_019356796.2 |
| ***atg8b*** | GCCTCCAGCTAAACTCCAACC | CGCTCTCGCTCGTACACCTC | XM_003439438.5 |
| ***p62*** | GCTCAGAACCAGGACAACGA | CATCTCCTCATCGCCCTCAC | XM_005463795.4 |
| ***ampkα1*** | CACAGTGGACAGCAGAACCT | GTGGGGTGCACAGTACTAGG | NM_001319868.1 |
| ***ampkα2*** | GGATGAAGAGGCTGTCAGGG | GAATTCACTGGCCTGGGTCA | XM_025902635.1 |
| ***ampkβ1*** | TCCTTCAACAACTGGGCCAA | CTGTGCCTAGCTGACTGGTT | XM_003440104.4 |
| ***ampkβ2*** | GACAGCACAGATGACCCCAA | TAGACCTCCTTCCCACCTCC | XM_005476192.4 |
| ***ampkγ1*** | CAGACACACCGCTCTACACA | TTGGTCACCGTCACATCCAG | XM_003441301.5 |
| ***ampkγ2*** | AACAAAATTCACCGGCTGCC | TATGTTCCGATGCCCAGCTC | [XM_013269492.3](https://www.ncbi.nlm.nih.gov/nucleotide/XM_013269492.3?report=genbank&log$=nucltop&blast_rank=1&RID=HW17TCUX016) |
| ***acads*** | CGCTAAACAGATTCAAGAGCTGG | TTCTTCCATAGCCAGACTGTACG | XM_013264734.3 |
| ***acadm*** | CCCTTGGCTTCTCATTTGAGTTG | ACCACTCTTGTCATAAGCAGGAG | XM_003448445.4 |
| ***acadvl*** | GGTAAATGATCCAGCCAAGAACG | TTACTCCCAACGATCTCAACGAG | XM_003458877.5 |
| ***acadl*** | AAAGACTGAGATCTGTGTGGGTC | CTCGTTGGTTCCTCCGTAGATAG | XM_003453184.3 |
| ***acadsb*** | GCTGTTTGTTAAACTCGGTACCC | CTGTATCCAGCAGAGAG ATCCAC | XM_019362513 |
| ***acox1*** | CCACTATAAGAGGCACACACCTT | CAGTGGGACTGTTCAAGACAAAC | XM_019359415.2 |
| ***acox3*** | AGGAGTCTCAGGAGATCCTCAAA | CCAGTGACCTGCAGTAGTAAACT | XM_013266255 |
| ***acaa*** | TCTTTAACGTAGCAGGAGCCATC | GAATGATGCAGTCTCTGGCTTTG | XM_025908226.1 |
| ***hadh*** | ATCCTAACAACCCCCTCTTTGAC | CCTCTCCTGTCTTTATGCCAAGT | XM_003452225 |
| ***hadhb*** | CATGGCAAATCTGAAGGCTATGG | CAGTCTGTTTGCTACTGTGGTTG | XM_003446255.4 |
| ***β-actin*** | AGCCTTCCTTCCTTGGTATGGAAT | TGTTGGCGTACAGGTCCTTACG | KJ126772.1 |
| ***rpl7*** | TGCCCTCACAGACAATGCTT | GTCTTCTTGTTCATGCCGCC | XM_003443469.4 |
| ***hprt1*** | TCGGTGGAGATGACCTGTCT | TCTAGCGCATAACCCACCAC | XM_003455821.5 |
| ***b2m*** | CCCCTGACATCACCATCACA | CCGTGAGTGACTTGCAGGT | MK789001.1 |
| ***ubce*** | GAGGGAGGGGTCTTCTTCCT | GGACAGCAACACCTTGGAGA | XM_003443150.5 |
| ***18s*** | AGCCACACGAGATTGAGCAA | TGTGTACAAAGGGCAGGGAC | XR_003216134.1 |
| ***tuba*** | CAACATCAACCGCCTTAT | ATTTCAGCCACAGACAGC | XM_005451915.4 |
| ***Plasmid construction*** |  |  |  |
| **GFP-Beclin1** | ctagcgtttaaacttaagcttATGGAGGGCTCCAAAACGTC | gtaccgtcgactgcagaattcCTTATCGTCGTCATCCTTGTAATC |  |
| **His-AMPK** | ctagcgtttaaacttaagcttATGGCGACGGAAAAAGCG | aacgggccctctagactcgagCTAATGGTGATGGTGATGATGTCG |  |
| ***siRNA sequences*** |  |  |  |
| **AMPKα1-siRNA**  **-1374** | GCUUGCCAAGAUGAGCCUUTT | AAGGCUCAUCUUGGCAAGCTT |  |
| **Beclin1-siRNA**  **-774** | GCUCGACACAGAGGAGCUATT | UAGCUCCUCUGUGUCGAGCTT |  |
| **NC- siRNA** | UUCUCCGAACGUGUCACGUTT | ACGUGACACGUUCGGAGAATT |  |

Abbreviations: *fas*, fatty acid synthase；*6pgd*, 6-phosphogluconate dehydrogenase; *g6pd*, glucose-6-phosphate 1-dehydrogenase; *g6pd*, glucose-6-phosphate dehydrogenase; *icdh*, isocitrate dehydrogenase1; *me*, malic enzyme; *scd-1*, stearoyl-Coenzyme A desaturase 1; *pparα*, peroxisome proliferator-activated receptor α; *srebp-1*, sterol regulatory element binding transcription factor 1; *beclin1*, Autophagy-related 6; *atg*, autophagy related gene; *lc3b*, microtubule associated protein 1 light chain 3 beta; *ampk*, AMP-activated protein kinase; *acads*, acyl-CoA dehydrogenase short chain; *acadm*, acyl-CoA dehydrogenase medium chain; *acadvl*, acyl-CoA dehydrogenase very long chain; *acadl*, acyl-CoA dehydrogenase long chain; *acadsb*, acyl-CoA dehydrogenase short/branched chain; *acox*, acyl-CoA oxidase; *acaa*, acetyl-CoA acyltransferase 1; *hadh*, hydroxyacyl-CoA dehydrogenase; *hadhb*, hydroxyacyl-CoA dehydrogenase trifunctional multienzyme complex subunit beta. *rpl7*, ribosomal protein l7; *hprt1*, hypoxanthine phosphoribosyltransferase 1; *tuba*, tubulin alpha chain; *b2m*, beta-2-microglobulin; *ubce*, ubiquitin-conjugating enzyme; *18s*, 18s ribosomal RNA;

***Supplementary Table 4*** *Effects of dietary phosphorus supplementation on growth performance and morphological parameters of tilapia after 10 weeks.*

|  | LDP | MDP | HDP |
| --- | --- | --- | --- |
| IBW (g/fish) | 8.86±0.01 | 8.87±0.01 | 8.91±0.00 |
| FBW (g/fish) | 66.0±1.7^a^ | 84.0±0.3^b^ | 92.8±3.3^c^ |
| WG^1^ (%) | 645.6±18.5^a^ | 846.6±4.8^b^ | 941.6±37.0^c^ |
| SGR^2^ (%/d) | 2.87±0.04^a^ | 3.21±0.00^b^ | 3.35±0.05^c^ |
| FI^3^, g/fish | 69.15±20.57 ^a^ | 79.65±0.47 ^b^ | 84.73±1.04 ^b^ |
| FCR^4^ | 1.60±0.07^b^ | 1.32±0.01^b^ | 1.24±0.06^a^ |
| HSI^5^ (%) | 2.27±0.14 | 2.50±0.09 | 2.16±0.11 |
| CF^6^ (%) | 3.43±0.08 ^a^ | 3.44±0.23 ^ab^ | 3.64±0.09 ^b^ |
| Survival^7^ (%) | 92.00±0.02 | 93.33±0.01 | 94.67±0.13 |

IBW, Initial mean body weight; FBW, final mean body weight; WG, weight gain; SGR, specific growth rate; FCR, feed conversion rate; HSI: hepatosomatic index; CF, condition factor; FI, feed intake.

Values are mean ± SEM (n=3 replicate tanks. IBW, FBW, WG, SGR, FCR: replicates of 25 fish). “a b c” indicate significant difference among the three groups, as determined by one-way ANOVA, and further post hoc Duncan’s multiple range testing. *P*≤0.05.

^1^WG = (FBW-IBW)/IBW×100;

^2^SGR = 100×[ln (FBW)-ln (IBW)]/d;

^3^FI=dry feed fed (g)/fish numbers;

^4^FCR = dry food fed (g)/wet weight gain (g);

^5^HSI = 100×(liver weight)/(body weight);

^6^CF = 100×(live weight, g)/(body length, cm)^3^;

^7^Survival= 100 ×final fish number/initial fish number.


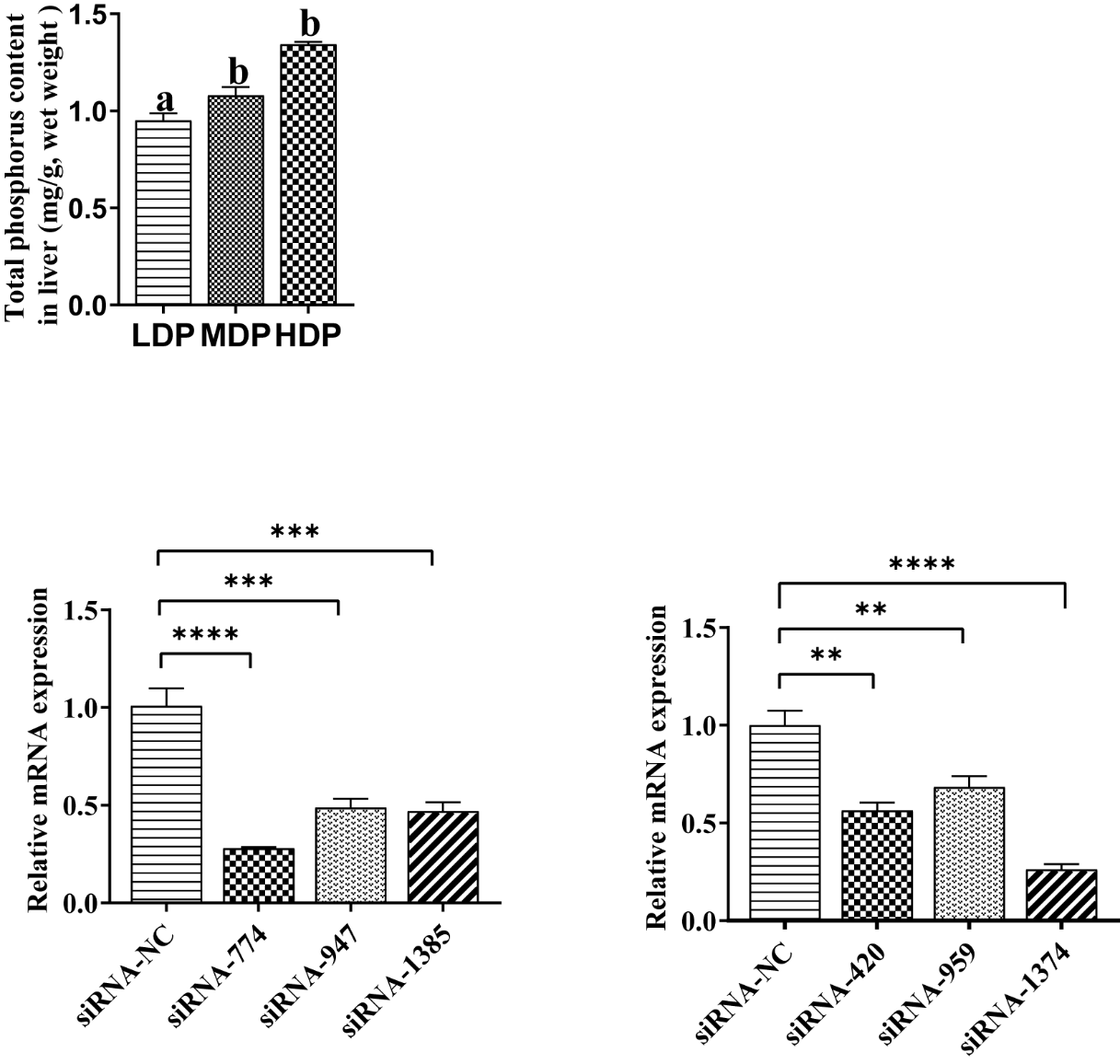


**Supplementary Figure 1** Hepatic total phosphorus content in the liver of GIFT tilapia. Data are mean ± SEM (n=3). “a-c” denote significance at *P*≤0.05. The *P* Value was calculated by one-way ANOVA and further post hoc Duncan’s multiple range testing.


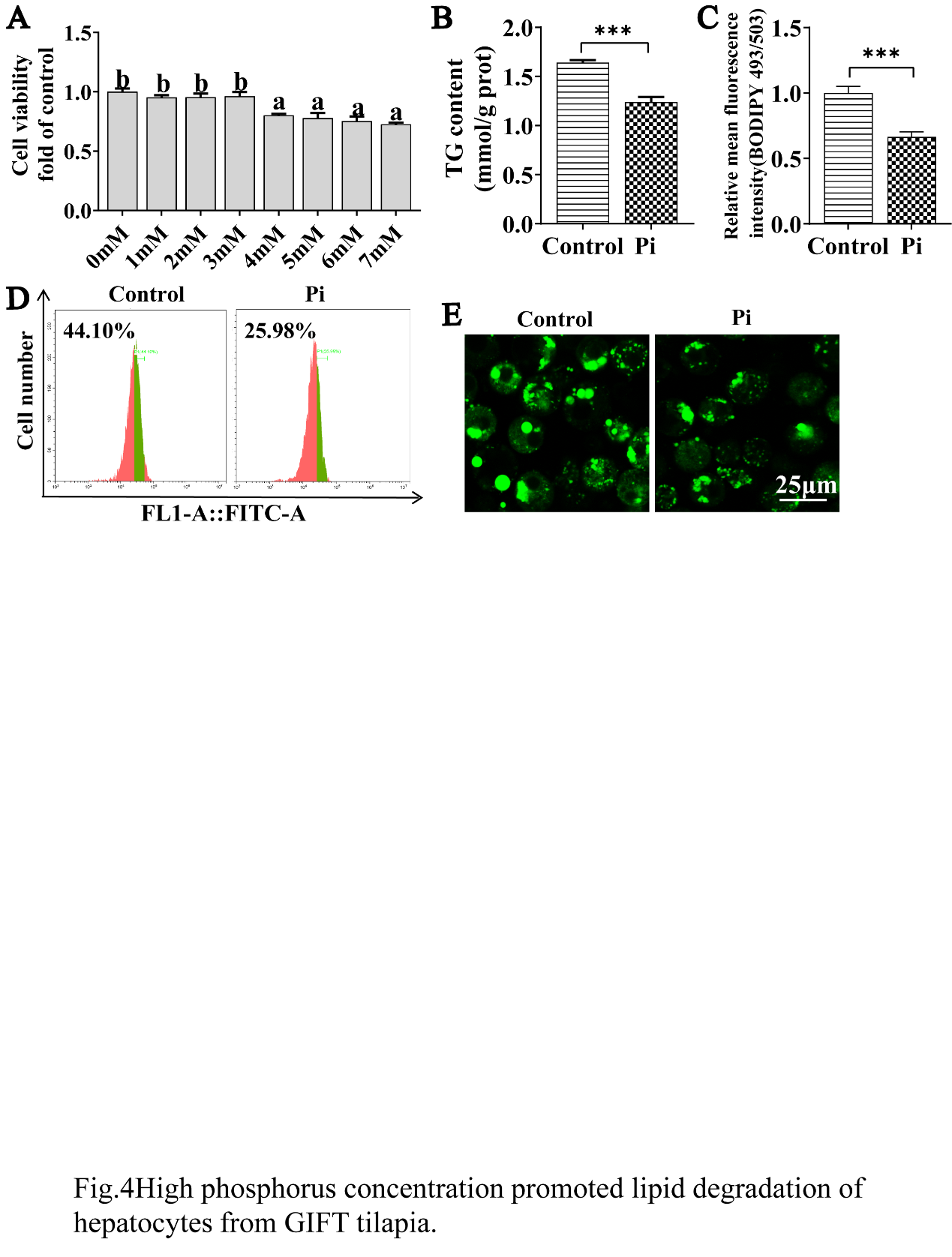


**Supplementary Figure 2** Pi reduces lipid content in tilapia hepatocytes for 48h in L-15 medium. **(A)** MTT assay of Pi. **(B)** TG content. **(C)** The presence of LDs with Bodipy 493/503 staining demonstrated by flow cytometry of the primary hepatocytes of GIFT tilapia. **(D)** The quantification of Bodipy 493/503-stained LDs by flow cytometric analysis of FL1 (green) mean fluorescence intensity of primary hepatocytes of GIFT tilapia. **(E)** Representative confocal microscopy image analysis of primary hepatocytes of GIFT tilapia with Bodipy 493/503 staining. Scale bar, 25μm.


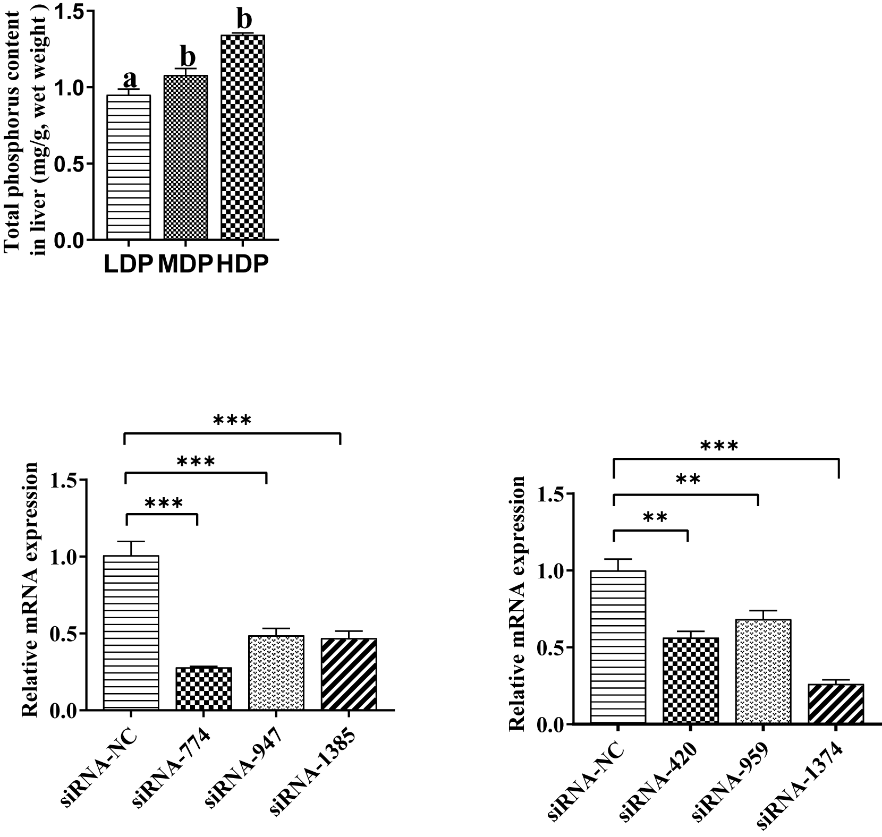


**Supplementary** **Figure 3** The siRNA knockdown efficiency of *beclin1* gene in tilapia hepatocytes for 48h in L-15 medium. Data are mean ± SEM (n=3), **P*≤0.05, ***P*≤0.01, ****P*≤0.001, as determined by one-way ANOVA with the Bonferroni posthoc test.


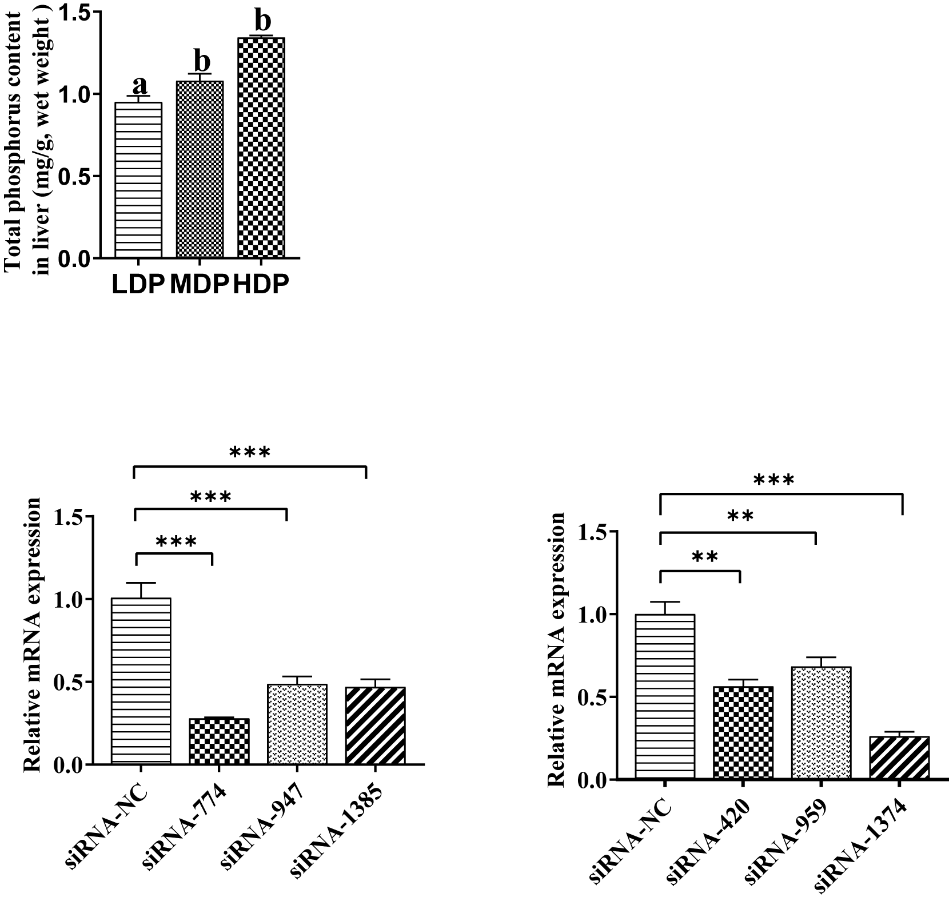


**Supplementary** **Figure 4** The siRNA knockdown efficiency of *ampkα1* gene in tilapia hepatocytes for 48h in L-15 medium. Data are mean ± SEM (n=3), **P*≤0.05, ***P*≤0.01, ****P*≤0.001, as determined by one-way ANOVA with the Bonferroni posthoc test.


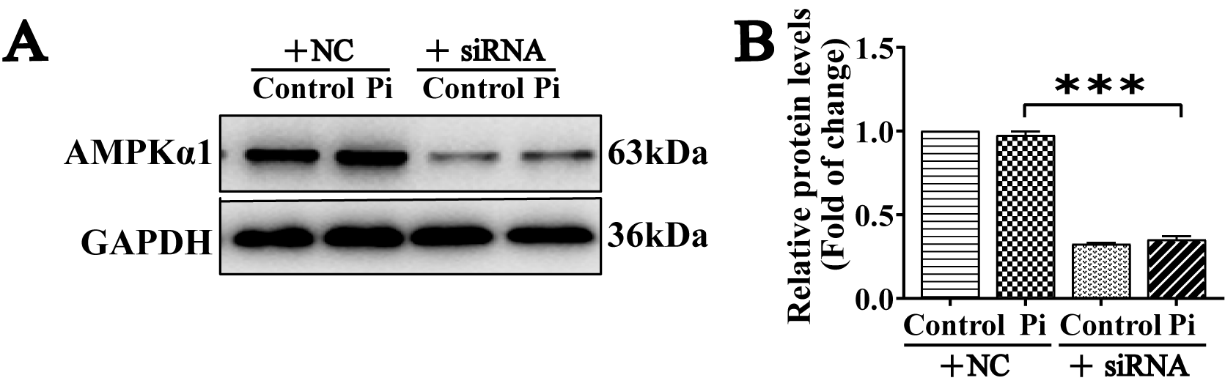


**Supplementary Figure 5** The siRNA knockdown efficiency of *ampkα1* gene in tilapia hepatocytes for 48h in L-15 medium. (A-B) Western blot analysis of AMPKα1 protein levels. Data are mean ± SEM (n=3), **P*≤0.05, ***P*≤0.01, ****P*≤0.001, as determined by one-way ANOVA with the Bonferroni posthoc test.
